# Cholesterol-Secreting and Statin-Responsive Hepatocytes from Human ES and iPS Cells to Model Hepatic Involvement in Cardiovascular Health

**DOI:** 10.1371/journal.pone.0067296

**Published:** 2013-07-11

**Authors:** Winfried H. Krueger, Borko Tanasijevic, Vanessa Barber, Anthony Flamier, Xinsheng Gu, Jose Manautou, Theodore P. Rasmussen

**Affiliations:** 1 Department of Pharmaceutical Sciences, University of Connecticut, Storrs, Connecticut, United States of America; 2 University of Connecticut Stem Cell Institute, Farmington, Connecticut, United States of America; 3 Department of Molecular and Cell Biology, University of Connecticut, Storrs, Connecticut, United States of America; University of Kansas Medical Center, United States of America

## Abstract

Hepatocytes play a central and crucial role in cholesterol and lipid homeostasis, and their proper function is of key importance for cardiovascular health. In particular, hepatocytes (especially periportal hepatocytes) endogenously synthesize large amounts of cholesterol and secrete it into circulating blood via apolipoprotein particles. Cholesterol-secreting hepatocytes are also the clinically-relevant cells targeted by statin treatment *in vivo*. The study of cholesterol homeostasis is largely restricted to the use of animal models and immortalized cell lines that do not recapitulate those key aspects of normal human hepatocyte function that result from genetic variation of individuals within a population. Hepatocyte-like cells (HLCs) derived from human embryonic and induced pluripotent stem cells can provide a cell culture model for the study of cholesterol homeostasis, dyslipidemias, the action of statins and other pharmaceuticals important for cardiovascular health. We have analyzed expression of core components for cholesterol homeostasis in untreated human iPS cells and in response to pravastatin. Here we show the production of differentiated cells resembling periportal hepatocytes from human pluripotent stem cells. These cells express a broad range of apolipoproteins required for secretion and elimination of serum cholesterol, actively secrete cholesterol into the medium, and respond functionally to statin treatment by reduced cholesterol secretion. Our research shows that HLCs derived from human pluripotent cells provide a robust cell culture system for the investigation of the hepatic contribution to human cholesterol homeostasis at both cellular and molecular levels. Importantly, it permits for the first time to also functionally assess the impact of genetic polymorphisms on cholesterol homeostasis. Finally, the system will also be useful for mechanistic studies of heritable dyslipidemias, drug discovery, and investigation of modes of action of cholesterol-modulatory drugs.

## Introduction

Chronic exposure to high serum cholesterol is the single largest risk factor for the development of atherosclerosis and results from perturbed cholesterol homeostasis [1]. Hepatocytes produce more than 20% of endogenously-synthesized cholesterol in the body, and only hepatocytes secrete endogenously-synthesized cholesterol into the blood [2]. Serum cholesterol of both dietary and endogenous origin is ferried to its destinations by chylomicrons, high, low, and very low density lipoprotein particles (HDL, i.e. “good cholesterol, LDL, i.e. bad cholesterol” and VLDL). HDL mediates reverse cholesterol transport, the process whereby excess serum cholesterol is removed from the blood [3] and increased levels of HDL correlate with reduced risk of atherosclerosis [4]. In contrast, LDL and VLDL particles transport triglycerides and cholesterol from the liver to sites of usage throughout the body and elevated LDL levels also correlate with elevated risk of atherosclerosis. Thus, serum HDL/LDL ratios are clinically used as risk indicators for atheromatous plaque and cardiovascular disease (CVD) development.

Apolipoproteins (APOs), a family of more than 20 proteins, are key components of lipoprotein particles [5] and several are expressed predominantly in hepatocytes including the HDL components APOA1 and APOA2, the LDL and VLDL component APOB100, the VLDL component APOC3, and APOE, which is included in all lipoprotein particles. Mutations in HDL genes or decreased levels of serum HDL are associated with increased risk for atheromatous plaque development [6] as are mutations in the APOB100 gene [7]. Increased levels of APOC3 induce the development of hypertriglyceridemia [8] and APOE isoform imbalances are intimately linked to CVD [9], [10].

Within the liver, functionally-specialized hepatocytes are sequentially organized into periportal, mid-zonal, and perivenular zones along the sinusoidal tubules of the liver cell plate within liver lobules [11]. Hepatic blood first enters the periportal zone which contains hepatocytes that express high levels of HMGCoA reductase (HMGCR), the key enzyme involved in endogenous cholesterol biosynthesis [12]. Periportal hepatocytes also secrete and endocytose apolipoprotein particles [13], [14] including APOE, though this zonal distribution is predominantly confined to males [14]. In contrast, the highest levels of cytochrome P450 (CYP) enzymes, crucial for drug metabolism and detoxification are expressed in perivenular hepatocytes [11].Thus, periportal hepatocytes are especially crucial for cardiovascular health since they are actively involved in secretion of high levels of cholesterol and associated apolipoproteins into the blood.

Current model systems to study hepatocyte function include the use of primary human hepatocytes, animal models, and transformed hepatic cell lines. These models suffer from substantial and fundamental deficiencies. Primary hepatocytes are unstable in cell culture, and have variable and short-lived CYP activity [15]. Rodent models are costly and do not model human apolipoprotein profiles adequately. Lastly, immortalized hepatic cell lines such as HepG2 cells have little residual CYP activity, and are in general metabolically compromised.

In the current study we have reprogrammed skin-derived dermal fibroblasts from healthy human donors [16], [17] and then directed their differentiation through a multistep differentiation procedure to hepatocyte-like cells (HLCs) [18] using a method that is based on embryonic liver organogenesis [19], [20]. Our HLCs express definitive hepatocyte markers, express a wide variety of apolipoproteins, secrete soluble cholesterol, and respond to statin treatment. Our system opens the door to direct studies of hepatocellular mechanisms that regulate HDL and LDL production in a human cell culture system, and provides a novel platform for the discovery, optimization, and investigation of modes of action of drugs designed to prevent or reverse atherosclerosis.

## Materials and Methods

### Cell culture

Primary human dermal fibroblasts were grown according to the vendor's instruction (hDF1: Montreal Cell bank: MCH065; hDF6: ATCC: catalog no. PCS-201-012) in MEM supplemented with 15% fetal bovine serum, 1x non-essential amino acids (NEAA, Invitrogen Inc.) and 2mM glutamine (Invitrogen Inc.). Cultures were passaged at 80-90% confluency. WA09 (H9) cells were obtained from WICELL at passage 19, adapted to growth on 35 μg/cm^2^ matrigel (BD Sciences Inc.) in mTESR1 (Stemcell Technologies Inc.). Pluripotent cells were maintained under above conditions throughout the experiment. Embryoid bodies were generated by transfer of mechanically dissociated stem cell colonies into culture plates coated with a 2% poly-2-hydroxyethyl-methacrylate (poly-hema) solution and cultured for 20 days in DMEM/F12 supplemented with 20% knock out serum replacement, 1x NEAA, 2mM glutamine and 100 μM β-mercaptoethanol. Hepatocytes were maintained on 35 μg/cm^2^ matrigel (BD Sciences Inc.) in hepatocyte growth medium (HCM, Promocell Technologies Inc.) supplemented with 10 ng/ml oncostatin M (Prospec Inc.) and dexamethasone (Sigma Inc.).

### Reprogramming of human dermal fibroblasts

Human dermal fibroblasts from 3 year (hDF1, Montreal Cell Bank) and 43 year (hDF6, ATCC) old healthy unaffected females were reprogrammed using a published protocol [17] with minor modifications. Briefly, reprogramming was induced by infecting 3×10^5^ fibroblasts either with a mixture of lentivirus particles (Openbiosystems Inc.) containing genomes encoding OCT4, SOX2, KLF4 and human c-MYC at a multiplicity of infection (moi) of 5∶5∶5∶2.5 (hDF6) or, for passage 13 cells (hDF1), with a similar mixture of lentivirus particles expressing the above genes (Addgene) and a LMNA shRNA at a moi of 5 in a doxycycline dependent fashion to create an ESC-like chromatin-state [21]. This approach yielded colonies with embryonic stem cell morphology at a frequency of 8×10^−5^ (data not shown).

Transduced fibroblasts were maintained in DMEM/10% FCS until day 4 post transduction. On day 3 post transduction, fibroblasts (100,000 cells per 100 mm tissue culture dish) were passaged onto feeder cells and on day 4 the medium was changed to embryonic stem cell culture medium consisting of DMEM/F12 (Invitrogen) containing 20% knock out serum replacement (Invitrogen), 1x non-essential amino acids, 1mM glutamine, 100 μM β-mercaptoethanol and 5 ng/ml FGF2. Media were changed daily and in the case of doxycycline-inducible expression of reprogramming factors, doxycycline was administered daily at a concentration of 2 μg/ml until day 18 post transduction. Candidate iPS cells were identified by colony morphology and transferred into fresh culture wells after 25 (hDF1) and 30 days (hDF6), respectively and maintained under feeder free conditions on matrigel in the presence of mTESR1. Viral titers were obtained by transduction at defined dilutions of the individual lentivirus into sentinel fibroblasts through immunofluorescent detection of the respective reprogramming factor.

### Fluorescence activated cell sorting of pluripotent stem cells

Cells were harvested at 70% of confluence and a single cell suspension was prepared by dissociation with Accutase (Invitrogen) for 5 min at 37°C and filtering through a 40μm cell strainer (BD Biosciences). Cells were then fixed with 4% paraformaldehyde for 15 min at room temperature, washed once with PBS and resuspended in a staining buffer (0.2%BSA in PBS). After 30 min on ice cells were incubated with anti-SSEA4 antibody conjugated to phycoerythericin and anti-Tra1-60 antibody conjugated to fluorescein (BD Biosciences Inc.). The cells are then washed two times in PBS and then resuspended in staining buffer at a concentration of 10^6^ cells/ml. For the compensation between FITC and PE, anti-mouse IgG BD CompBeads Plus (BD Biosciences Inc.) were used. About 2×10^4^ events (cells) were sorted using a FACS Calibur flow cytometer. FACS profiles were analyzed using CellQuest Pro Software (BD Biosciences Inc.) and FlowJo software v9 (Tree Star Inc.).

### Differentiation into Embryoid bodies

ES/iPS cells were grown in mTESR1 on matrigel until colonies of pluripotent cells had a diameter of approximately 1–2 mm before processing for embryoid body (EB) differentiation. Colonies were cut into smaller fragments and then dislodged mechanically. An average of 100 cell clumps were then transferred into ultralow adhesion plates (Becton Dickinson) and cultured for 21 days in DMEM/F12 supplemented with 20% knock-out serum replacement (Invitrogen Inc.), non-essential aminoacids (Invitrogen Inc.) and 2 mM glutamax (Invitrogen Inc.), 0.5 ng/ml FGF2 and 0.1 mM β-mercaptoethanol. Embryoid bodies (EBs) were then harvested directly into RLT (Qiagen Inc.) for preparation of total RNA.

### Germlayer differentiation

Pluripotent stem cells were differentiated into derivatives of ecto-, meso-, and endodermal lineage cells using the Human Pluripotent Stem Cell Functional Identification kit according to the manufacturer's instructions (R&D Systems). Briefly, cells were harvested as a single-cell suspension and plated onto matrigel at a density of 10^5^ cells/cm^2^. The cells were grown to approximately 50% confluency in mTeSR1 media (StemCell Technologies) and then differentiated into the three germ layers by replacing mTeSR1 media with lineage specific media (R&D Systems). After 4 days of culture, the cells were analyzed for Sox17 (endoderm), Otx2 (ectoderm) or Brachyury (mesoderm) protein expression by immunofluorescence.

### Hepatocyte differentiation

ES/iPS cells were grown in mTESR1 on matrigel until colonies of pluripotent cells had a diameter of approximately 1–2 mmbefore differentiation was initiated. Differentiation was performed by methods similar to those previously described [18], [22]. Briefly, definitive endoderm was produced through culture in induction medium (RPMI 1640 containing 0.3% bovine serum albumin, 1x non-essential amino acids (Invitrogen), 2 mM glutamine (Invitrogen Inc.) and 100 ng/ml Activin A (Prospec Inc.). On day 2 the culture medium was replaced with fresh induction medium containing 0.1x insulin transferrin selenium complexes (ITS, Sigma) and on day 3 the culture medium was replaced with fresh induction medium containing 1x ITS. Hepatocellular lineages were induced on day 4 with HCM containing 20 ng/ml BMP4 (Prospec Inc.) and 10 ng/ml FGF2 (R&D Systems) (stage 2 medium) for five days. On day 9, hepatoblasts were switched to HCM containing 20 ng/ml HGF (Peprotech Inc.) (stage 3A medium) and cultured for five days. Hepatocyte-like cells (stage 3B cells) were generated through culture for 5 days in HCM containing 10 ng/ml OSM and 0.1 μM Dexamethasone (stage 3B medium) until harvest of the cells for analysis. For measuring secreted cholesterol the phenol red-free variant of HCM was used.

### Pravastatin treatment and cholesterol assays

Stage 3B HLCs were cultured for 48 hours in HCM containing 10 μM pravastatin (Sigma Inc.). Culture medium was retained, cleared of cellular debris by centrifugation at 14,300× g and an aliquot was assayed for soluble cholesterol content using the Amplex Red Cholesterol assay kit according to the manufacturer's instructions (Invitrogen Inc.). Cholesterol amounts were normalized to total DNA content of adherent cells.

### RNA extraction

RNA was prepared in triplicates from biologically independent samples. Cells were lysed in RLT buffer (Qiagen Inc.) containing 1% β-mercaptoethanol and processed using RNeasy ion exchange column chromatography according to the manufacturer's instructions (Qiagen Inc.). Purified RNA was quantified by UV spectroscopy.

### DNA extraction

DNA was prepared in triplicates from biologically independent samples. Cell pellets were resuspended in digestion buffer (100 mM NaCl, 50 mM Tris-HCl, pH8 and 5 mM EDTA) supplemented with 60 μg/ml DNase-free RNase and incubated at RT for 10 min. DNA was released overnight at 56°C in the presence of 0.5% SDS and Proteinase K (100μg/ml) and peptides were removed by phenol extraction using phase lock gels according to the manufacturer's instructions (Qiagen Inc.). DNA was quantified by UV spectroscopy.

### qRT-PCR

An amount of 500 ng of total RNA and the iScript^TM^ cDNA synthesis kit (BioRad) was used for cDNA synthesis. Quantitative real-time PCR analyses were performed using the iTaq Fast SYBR Green Supermix with ROX (BioRad) and a 7500 Fast Real Time PCR System (Applied Biosystems). Data were analysed by means of 2^−ΔCt^ method for relative quantification [23], using β-actin mRNA levels as endogenous reference. Primers were designed using the Primer Express software (Applied Biosystems) and are listed in Table S1. All samples were analysed in triplicates from biologically independent samples.

### Immunofluorescence

Antibodies used in this study included polyclonal anti-human albumin antibodies (rabbit, Dako; goat, Novus), monoclonal anti-human-AFP, -APOA1, -APOA2, -APOC3 and -LDLR antibodies (rabbit, Novus), a monoclonal anti-human SOX17 antibody (mouse, Novus), a biotinylated human TRA1-60 antibody (Cell Signaling) and a polyclonal anti-human NANOG antibody (Millipore). For immunofluorescent detection of the antigens, primary antibodies were diluted 1:200 and Alexa 488 and Alexa 594 coupled secondary antibodies (Invitrogen) 1:1000. Cells were fixed in 4% paraformaldehyde, washed three times in TBS (150 mM NaCl, 2.7 mM KCl, 20 mM Tris-HCl, pH 7.2) containing 1% bovine serum albumin (BSA), permeabilized in 0.1% Triton X-100, blocked in TBS containing 3% BSA and incubated with the appropriate primary antibodies in TBS containing 3% BSA for a minimum of two hours. After three washes cells were incubated with the appropriate secondary antibodies in TBS containing 3% BSA for a minimum of two hours, washed three times and imaged. Nuclei were marked using TBS containing 1% BSA and 4ng/ml of 4′, 6′-diamidino-2-phenylindole (Invitrogen Inc.). For quantitative immunofluorescence 2000 fixed cells were spun onto poly-lysine coated coverslips and processed as described above. A minimum of 200 cells per set was scored for antigen positivity and negativity and expressed proportionally for graphical representation. Proportions were determined by dividing the number of cells counted with a given pattern by the total number of cells (n) and all samples were analysed in triplicates from biologically independent samples. The standard deviation was determined using the equation s × sqrt(p(1-p)/n), where p is the proportion under consideration and n is the total number of cells counted.

## Results

### Generation and characterization of human pluripotent stem cells

**Figure 1:**
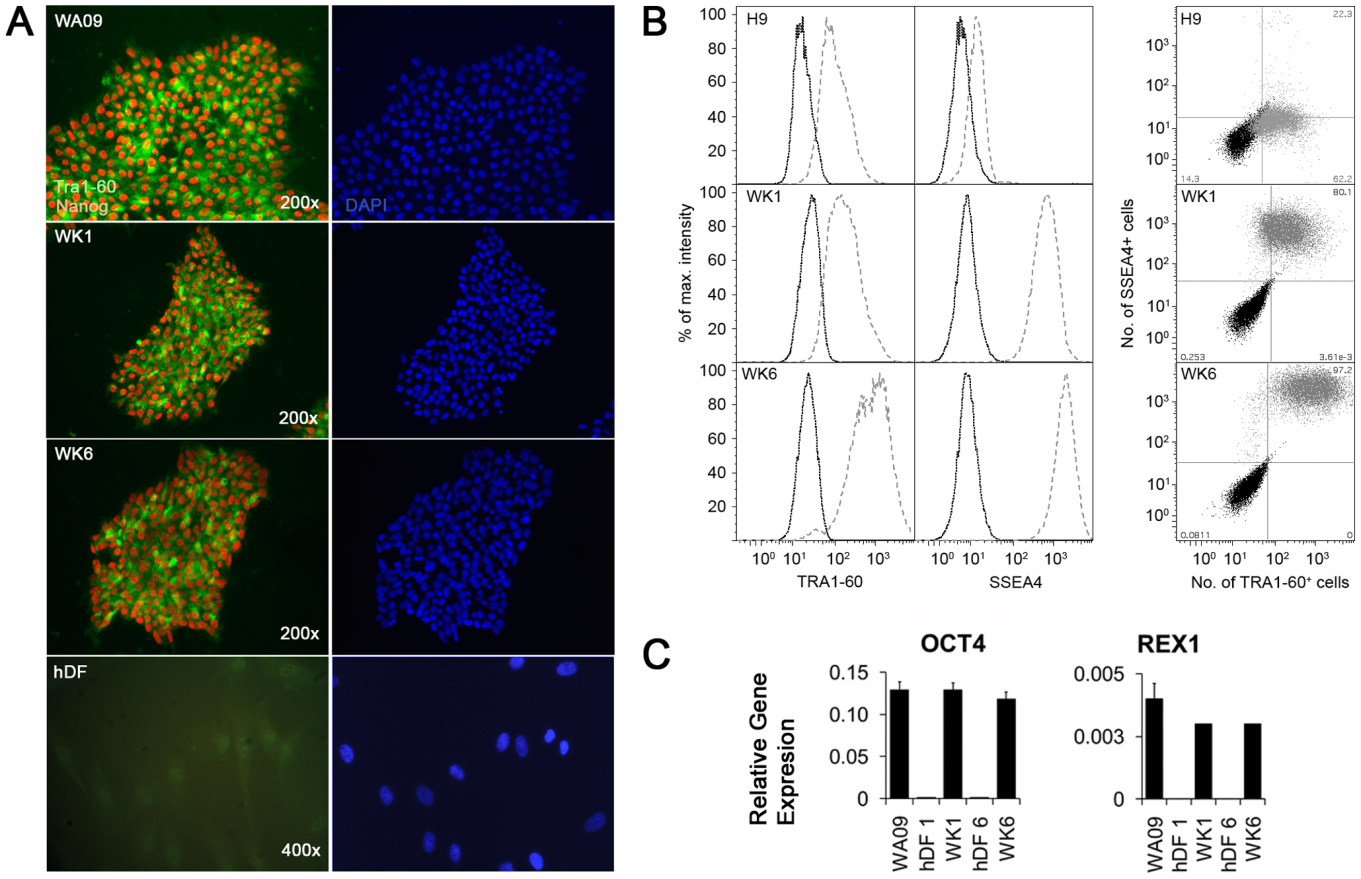
Reprogramming of normal human dermal fibroblasts. (A) Expression of the pluripotency markers NANOG and TRA1-60 as detected by immunofluorescence. Expression of NANOG (red) and TRA1-60 (green) was analysed in human embryonic stem cells (WA09) and human induced pluripotent stem cells (WK1 and WK6). Also shown is hDF1, the primary dermal fibroblast line that yielded WK1 upon OCT4, SOX2, KLF4 and c-MYC- mediated iPS. (B) Quantitative analysis by flow cytometry of Tra1-60 and SSEA4 cell surface marker expression in WA09, WK1 and WK6 cells. Cell were grown under feeder free conditions and cell surface markers detected using fluorescein-conjugated anti-human Tra1-60 and phycoerytherin-conjugated anti-human SSEA4 as described in the materials and methods before sorting. For graphical display, cell numbers for each bin were normalized to the bin containing the highest number of cells and plotted as a function of their relative fluorescence intensity (RFU). Histograms show both the untreated control (dotted line) and the treated cells (dashed line). (C) Quantitative analysis by flow cytometry of Tra1-60 and SSEA4 cell surface marker co-expression in WA09, WK1 and WK6 cells. Relative fluorescence intensities for SSEA4 are displayed as a function of the relative fluorescence intensities for Tra1-60 in the all cells. Untreated cells, (black dots) are displayed for identification of “non-fluorescent” cells, treated cells (grey dots) were identified through exclusion of non-fluorescent cells through gating into “non-fluorescent”, Tra1-60+ cells, SSEA4+ cells and Tra1-60+/SSEA4+ double positive cells. The numbers in each corner show the percentage of stained cells in the four gates. (D) Analysis of mRNA levels for the pluripotency markers OCT4 and REX1 in human pluripotent stem cells by qRT-PCR. No significant differences among the three pluripotent stem cell lines were observed. Error bars represent the standard error of the mean.

**Figure 2:**
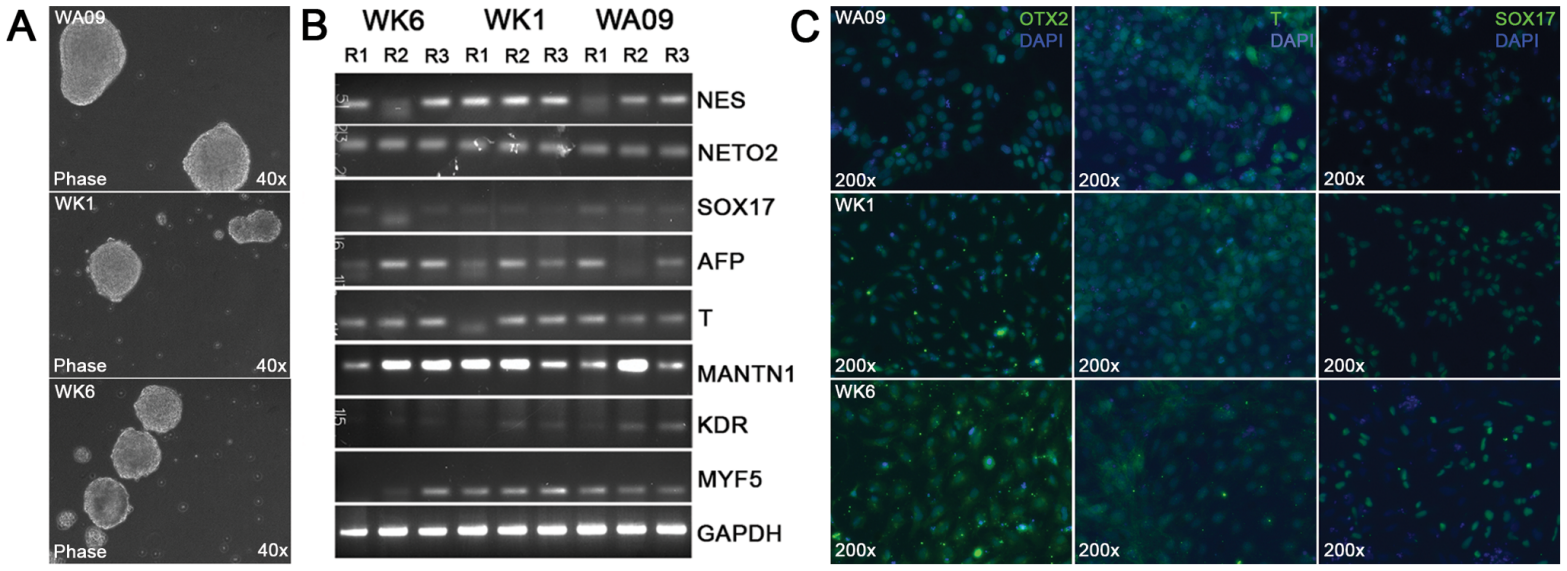
Pluripotency analysis of human pluripotent cells by embryoid body and directed differentiation. (A) Spontaneous differentiation of human pluripotent stem cells through EB formation. Pluripotent stem cells (WA09, WK1 and WK6) were seeded onto ultra-low binding substrates (Becton Dickinson) and assayed for aggregation (see experimental procedures). Human iPS cells aggregated to EBs with a tempo and size comparable to EBs derived from WA09 hESCs. (B) Analysis by semi-quantitative RT-PCR of germ layer marker expression by EBs derived from WA09, WK1 and WK6 cells. EB populations were grown in triplicates and harvested after 21 days of aggregation for the preparation of single stranded cDNA from total RNA. Germ layer-specific markers were detected by PCR and subsequent agarose gel electrophoresis using primers for the neuroepithelial (ectoderm) markers NESTIN (NES) and NEUROPILIN 2 (NETO2), the endoderm markers SOX17 and α-FETOPROTEIN (AFP) and the mesoderm markers BRACHYURY (T), MATRILIN1 (MANTN1), KDR, and MYF5. GAPDH was used as a loading control. (C) Directed differentiation of human pluripotent cells into early endodermal, ectodermal and mesodermal cells as described (Materials and Methods). Expression of the early lineage markers OTX2 (ectoderm), BRACHYURY (T, mesoderm) and SOX17 (endoderm) was detected by immunofluorescence microscopy using fluorescein-conjugated secondary antibodies. Cell nuclei were detected using DAPI (blue).

We derived two normal female human induced pluripotent stem cell (hiPSC) lines (WK1 and WK6) by standard iPS methods from dermal fibroblasts from a 3 year old female (hDF1) and 42 year old female (hDF6) donor, respectively and confirmed their pluripotency through assessment of pluripotency marker expression by immunofluorescence (Fig. 1A), fluorescence activated cell sorting (FACS, Fig. 1B and 1C), and quantitative real-time PCR (qRT-PCR) (Fig. 1C). Moreover, we employed both embryoid body (Fig. 2A and B) and directed differentiation (Fig. 2C) based methods to further confirm the pluripotency of our iPSCs. The pluripotency markers NANOG and TRA1-60 were readily detected in our iPSCs by immunofluorescence (Fig. 1A). Moreover, subsequent FACS based analysis demonstrated high level expression of the surface pluripotency markers SSEA4 and Tra1-60 (Fig. 1B). Intriguingly, we detected consistently low SSEA4 levels in our embryonic WA09 stem cell line (Fig. 1C) when cultured in mTeSR1 whereas cells cultured on mouse embryonic fibroblasts (Mefs) showed increased SSEA4 levels (data not shown). The significance of this finding is not understood at present and is currently under further investigation. Both, karyotyping and gene expression in EBs of WA09 cells grown in the presence of mTESR1 did not reveal any abnormalities (data not shown). Therefore, the up-regulation of SSEA4 after passaging of WA09 cells grown in the presence of mTESR1 onto Mef feeder cells argues for a deficiency in the mTESR1/matrigel culture system that leads, perhaps epigenetically, to the silencing of the SSEA4 locus.

Analysis by qRT-PCR for the key pluripotency transcription factors OCT4 (Fig. 1D) and REX1 (Fig. 1D) revealed that they were expressed in our iPSCs at levels comparable to WA09 human embryonic stem cells (hESCs) whereas they were not detected in the “parental” primary fibroblast cell lines hDF1 and hDF6. Finally, our iPSCs grow in defined colonies on both feeder cells and matrigel (Fig. S1A) and their morphological appearance resembles closely the cell morphology of ESCs with a large ratio of nucleus to cytoplasm (Fig. S1B). Both iPSC lines readily formed EBs at a tempo and size comparable to embryonic stem cells (Fig. 2A) and semi-quantitative RT-PCR revealed that the EBs contained cells belonging to all three germ layer lineages (Fig. 2B) as evidenced through the detection of the neuroepithelial markers NESTIN/NES and Neuropilin 2/NETO2, the endoderm markers SOX17 and AFP and the mesoderm markers BRACHYURY/T, MATRILIN1/MANTN1, KDR and MYF5. Moreover, both WK1 and WK6 cells also produced cells of ecto-, meso-, and endodermal origin in a directed differentiation approach as evidenced through the expression of Otx2, Brachyury and Sox17 proteins, respectively (Fig. 2C). Finally, WK1 and WK6 cells also produced mature MAP2-positive neurons (Akbarian, personal communication) and HLCs (this study) through directed differentiation. Together, these data demonstrate the pluripotent behavior of WK1 and WK6 hiPSCs.

### Directed differentiation of human pluripotent stem cells into hepatocyte-like cells (HLCs)

**Figure 3:**
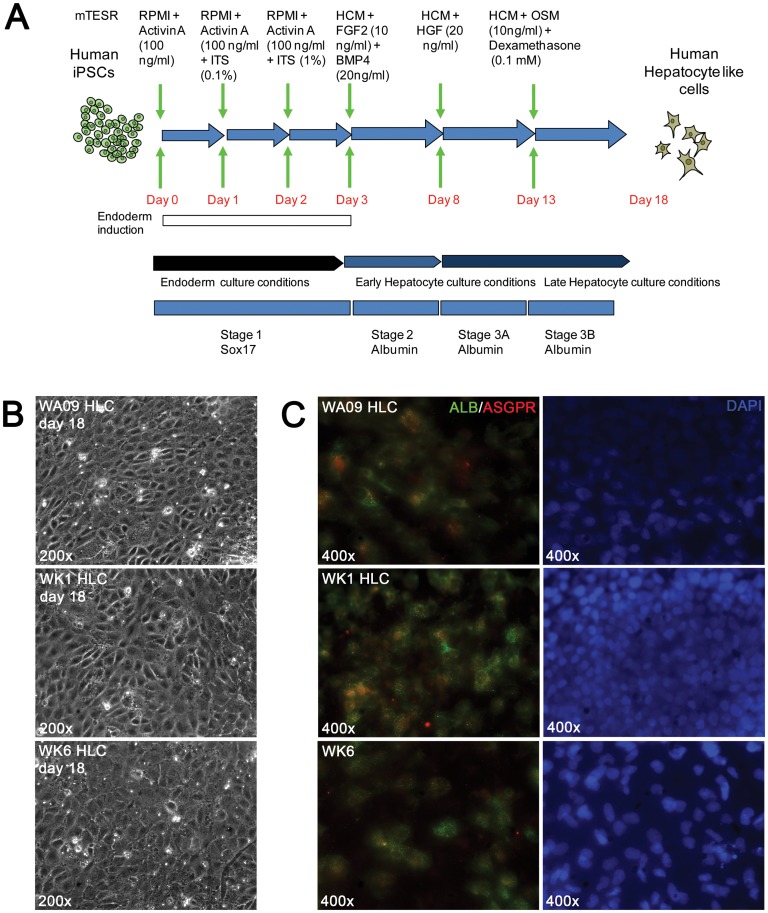
Directed differentiation of human pluripotent stem cells to hepatocyte-like cells. (A) Schematic representation of the directed differentiation procedure and time course. (B) Immunofluorescent assay for the definitive endoderm markers ALB and ASGPR in stage 3B HLCs derived from the hiPSC lines WA09, WK1 and WK6. See Fig. S1 for complete stage by stage analysis).

HLCs were generated from pluripotent stem cells cultured on matrigel in the presence of mTESR1 by the sequential exposure of pluripotent cells to cocktails of growth factors in chemically defined media (Fig. 3A). Human iPSCs exposed to Activin A differentiated into definitive endoderm within three days and expressed the definitive endoderm marker SOX17 (Fig. S2). The moderate levels observed may be due to side effects of signaling by insulin that is included in the later stages of endoderm induction to enhance cell survival. Subsequent exposure to fibroblast growth factor 2 (FGF2) and bone morphogenetic protein 4 (BMP4) induced expression of the definitive hepatocyte marker albumin (ALB) (Fig. S1) and the number of ALB expressing cells increased further upon switching to hepatocyte growth factor (HGF) (stage 3A). Further culture with dexamethasone (Dex) and oncostatin M (OSM) for an additional five days led to the appearance of large patches of cells with the polygonal morphology typical for hepatocytes (Fig. 3B) that also demonstrated an additional increase both in the intensity of the ALB fluorescence and the number of ALB positive cells at final stage 3B (Fig. 3C).

**Figure 4:**
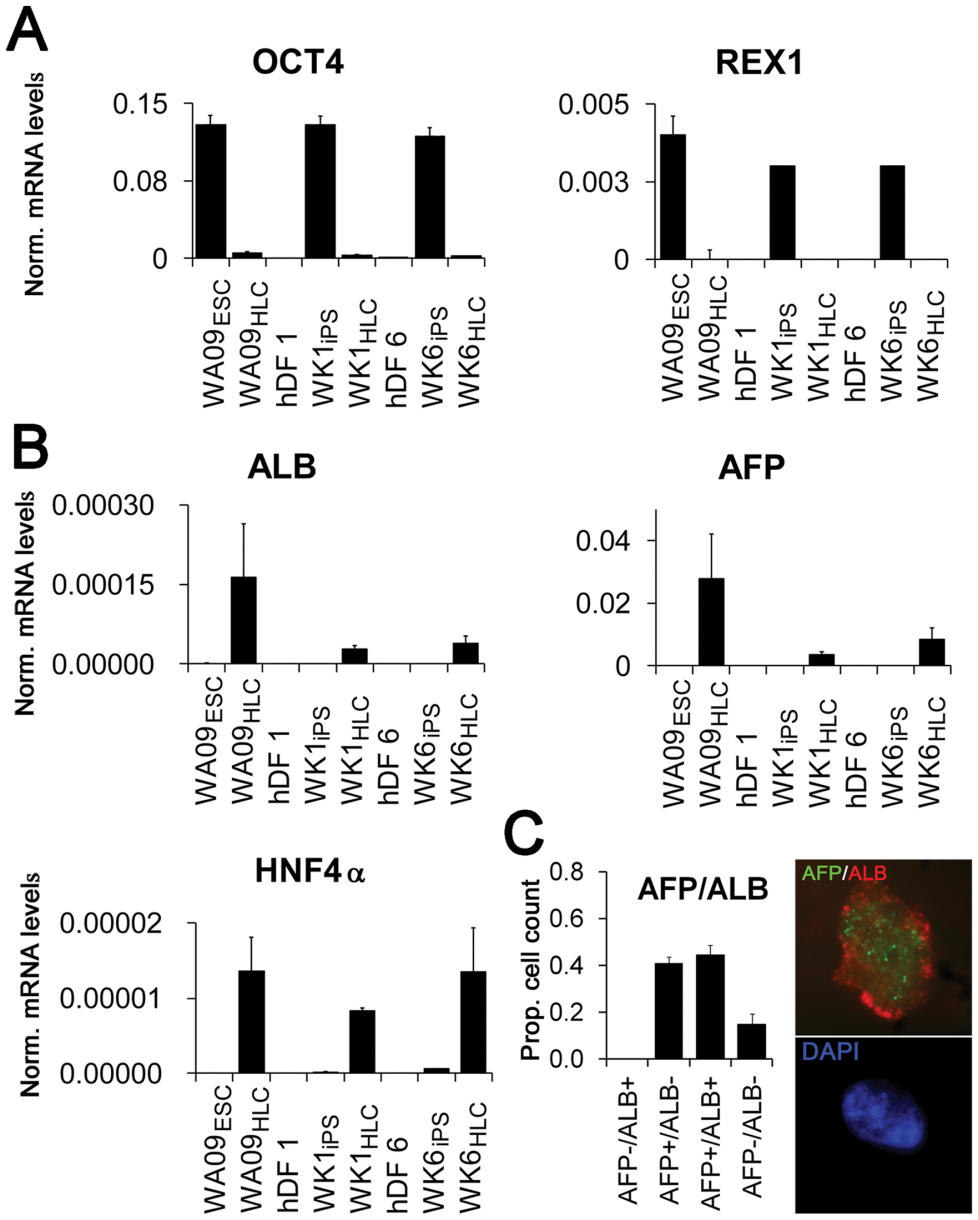
Expression of pluripotency and definitive hepatocyte markers before and after iPS, and in HLCs derived from pluripotent cells. (A) Analysis of expression of the pluripotency markers OCT4 and REX1 by qRT-PCR in: undifferentiated hESCs (WA09_ESC_) and stage 3B HLCs derived from WAO9 (WAO9_HLC_); dermal fibroblast line hDF1, hiPSC line WK1 derived from hDF1 (WK1_iPS_) and stage 3B hepatocyte-like cells derived from WK1 (WK1_HLC_); and dermal fibroblast line hDF6, the hiPSC line WK6 derived from hDF1 (WK1_iPS_) and stage 3B hepatocyte-like cells derived from WK6 (WK6_HLC_). (B) Induction of expression of the hepatocyte markers ALB, AFP, and HNF4α by qRT-PCR before and after reprogramming, and after stage 3B differentiation (for cell-type nomenclature see Fig. 3A legend). (C) Cell counts for AFP and ALB expression in cytocentrifuged stage 3B cells. WK1_HLCs_ were labeled with anti-human ALB and anti-human AFP antibodies and quantified as described (see Experimental Procedures). The inset depicts a representative high magnification image showing AFP (green) and ALB (red) expression in the top panel and DAPI (blue) in the bottom panel. Error bars represent the standard error of the mean.

We next assessed the quality of reprogramming and subsequent differentiation to stage 3B HLCs by qRT-PCR for pluripotency and hepatocyte-specific marker expression. Upon completion of the differentiation procedure, the pluripotency markers OCT4 and REX1 were robustly down-regulated in all HLCs (Fig. 4A) as expected. Hepatocyte-specific mRNAs were selected from 175 genes previously identified as highly expressed in liver cells [24] (Fig.4B, tables S2–S4). ALB, and α-fetoprotein (AFP), a marker for embryonic hepatocytes [25], were robustly up-regulated in HLCs derived from hESCs (WA09_HLC_) and hiPSCs (WK1_HLC_ and WK6_HLC_), though absolute expression levels of ALB and AFP mRNA in HLCs of hiPSC origin was lower than in HLCs of hESC origin (Fig. 4B). However, when compared to expression levels in the respective iPSCs, the up-regulation of AFP in WK6_HLCs_ and WA09_HLCs_ was similar (85,000 and 92,000 fold) whereas up-regulation in WK1_HLCs_ was less prominent (11,000 fold, data not shown). ALB levels were less induced, ranging from 90 fold for WK1_HLCs_ to 190 fold for WK6_HLCs_ and 400 fold for WA09_HLCs_ (data not shown). We also observed about 100 fold up-regulation of hepatocyte nuclear factor 4α (HNF4α), a nuclear receptor transcription factor that activates expression of numerous hepatocyte-specific genes encoding ALB, apolipoproteins, and several Cytochrome P450 (CYP) enzymes (Fig. 4B). Although these markers were up-regulated from two orders (HNF4A) to five orders of magnitude compared to pluripotent stem cells, their expression in our HLCs is still significantly lower than in HepG2 cells, primary hepatocytes and liver (Table 1).

**Table 1:** Comparative analysis of gene expression levels in HepG2 cells, primary hepatocytes, liver and HLC derived from WK1, WK6 and H9 cells.

	H9 HLC	WK1 HLC	WK6 HLC	HepG2	1° Hep.	Liver
CYP2E1	0.01107	0.00624	0.00876	0.17451	1	6397.53234
HNF4	0.0005	0.00034	0.00099	0.32113	1	2.02913
HMGCR	0.1625	0.1666	0.15494	0.15229	1	0.04894
ALB	0.00003	0.00001	0.00001	0.00636	1	0.35223
AFP	9153.05421	1139.33113	2809.72436	7585557.856	1	3.22954
APOA1	0.16837	0.08647	0.0838	0.05677	1	12.63807
APOA2	0.00043	0.00016	0.00023	0.00092	1	0.12791
APOA4	19.39736	2.79202	4.72439	1.06649	1	13.51947
APOB	0.00024	0.00002	0.00006	0.02875	1	0.10088
APOC3	0.0145	0.00515	0.00516	0.20603	1	53.53278
APOE	0.01166	0.00397	0.00657	0.04428	1	0.47982

Values for mRNA levels were expressed as fold β-actin mRNA amounts and then normalized to levels in primary hepatocytes.

Double-immunofluorescence for ALB and AFP expression in cytocentrifuged dissociated stage 3B cells (Fig. 4C) revealed that over 80% of the cells expressed AFP, ALB, or both proteins. About half of AFP expressing cells also expressed ALB, implying a progression from AFP+/ALB- to AFP+/ALB+ and AFP-/ALB+ cells and recapitulating *in vivo* data in which AFP expression commences in day 9.5 mouse embryos and declines dramatically in the mature liver, while ALB mRNA is first observed in e10.5 mouse embryos and reaches maximal levels in the mature liver [25]. We conclude our HLC cultures contain a mixture of early embryonic and mid-stage embryonic hepatocyte cell-types.

### APO expression in hepatocyte-like cells

**Figure 5:**
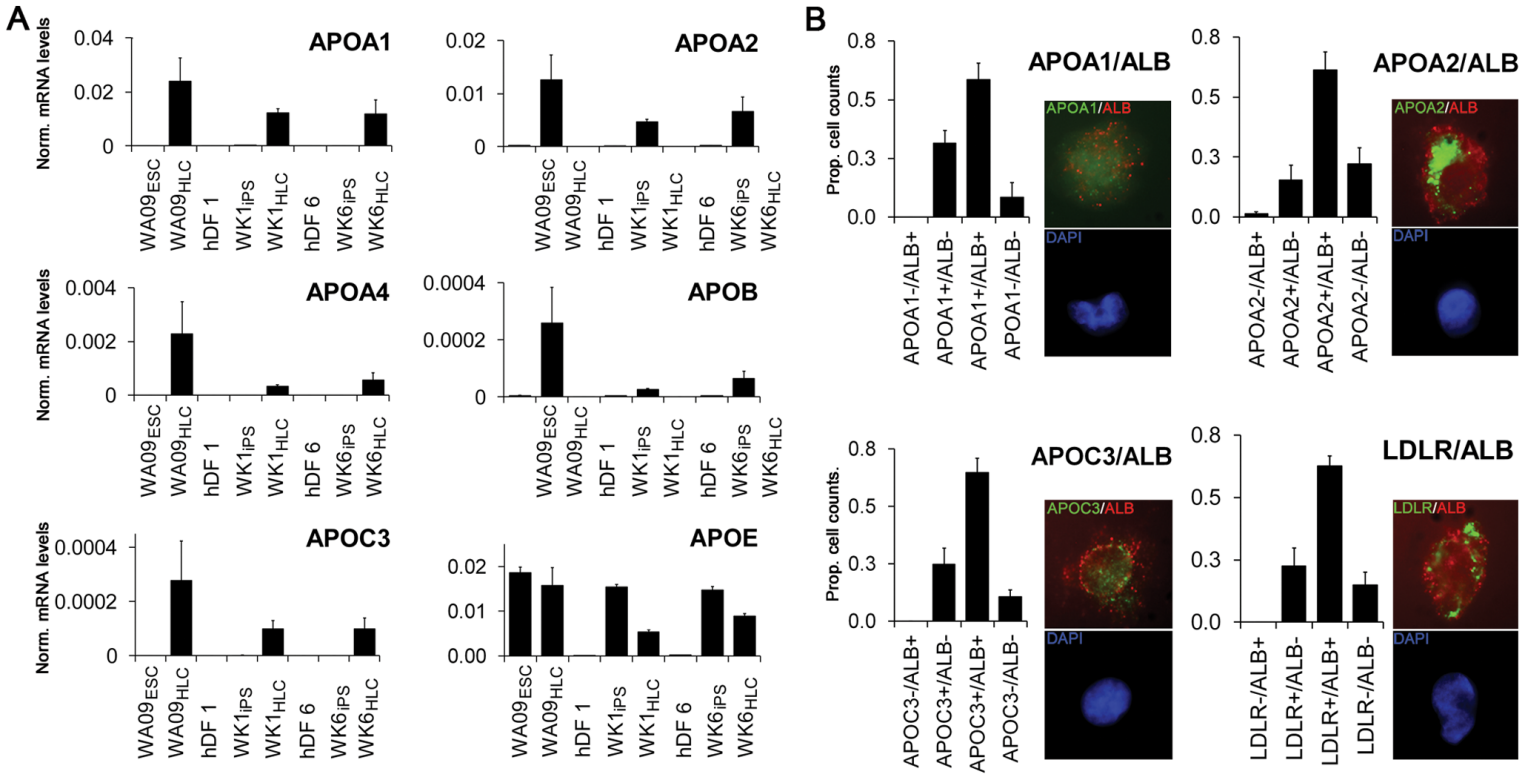
Induction of APO expression in HLCs derived from hESCs and hiPSCs. (A) Analysis of apolipoprotein A1, A2, A4, C3, E and LDLR mRNA expression by qRT-PCR (for cell-type nomenclature see Fig. 3A legend). Note that with the exception of APOB (LDL particles), APOC3 (VLDL particles) and APOE (all particles) all other apolipoproteins are part of HDL particles. Error bars represent the standard error of the mean. (B) Apolipoprotein expression by quantitative immunofluorescence. WK1_HLCs_ were labeled with anti-human ALB and either anti-human APOA1, APOA2, APOC3 or LDLR antibodies as described and analysed (see methods and materials). ALB expression was detected through a mouse anti-goat Alexa 594 conjugated secondary antibody (red) and the apolipoprotein expression was detected through a mouse anti-rabbit Alexa 488 conjugated secondary antibody. Insets depict representative high resolution images showing apolipoprotein (green) and albumin (red) expression in the top panels and DAPI (blue) in the bottom panels. Error bars represent the standard deviation.

Liver apoliproteins are key components for both release and uptake of serum cholesterol through formation of HDL, LDL and other lipoprotein particles. Messenger RNAs encoding several clinically-relevant apolipoproteins associated with HDL, LDL, IDL, VLDL, and chylomicrons were strongly up regulated in HLCs derived from WA09, WK1, and WK6 cells includingAPOA1 and APOA2, the principle apolipoproteins of HDL [26], [27], APOA4, a modulator of hepatic trans-cellular lipid transport found in HDL, VLDL, and chylomicrons [28], [29], [30], APOB, the major apolipoprotein component of LDL [31] and APOC3, the major apolipoprotein of VLDL [32] (Fig. 5A and tables S2–S4). We also found that APOE, expressed predominantly in periportal hepatocytes, was absent in dermal fibroblasts and was up-regulated in all three pluripotent cell lines upon differentiation to HLCs. APOE expression was observed in all three pluripotent cell lines consistent with a previous report of APOE expression in ES cells [33]. Notably, APOA1 expression was up to threefold higher in HLCs derived from iPSCs than in HepG2 cells but only one tenth of the amounts detected in primary hepatocytes. Remarkably, among all APO lipoproteins compared, APOA4 expression in our HLCs exceeded the amounts found in both HepG2 cells and primary hepatocytes and was comparable to levels detected in liver (Table 1).

Double immunofluorescence with dissociated cytocentrifuged stage 3B cells using antibodies specific for individual apolipoproteins in conjunction with an antibody for ALB showed significant co-expression of APOA1, APOA2, APOC3, and low density lipoprotein receptor (LDLR) with ALB in all stage 3B cultures (Fig. 5B). APOA1, APOA2, APOC3, and LDLR were also found to be expressed in a significant number of ALB-negative cells, and it is possible that these are AFP positive, but ALB negative, immature HLCs.

### Cholesterol secretion and pharmacology in hepatocyte-like cells

**Figure 6:**
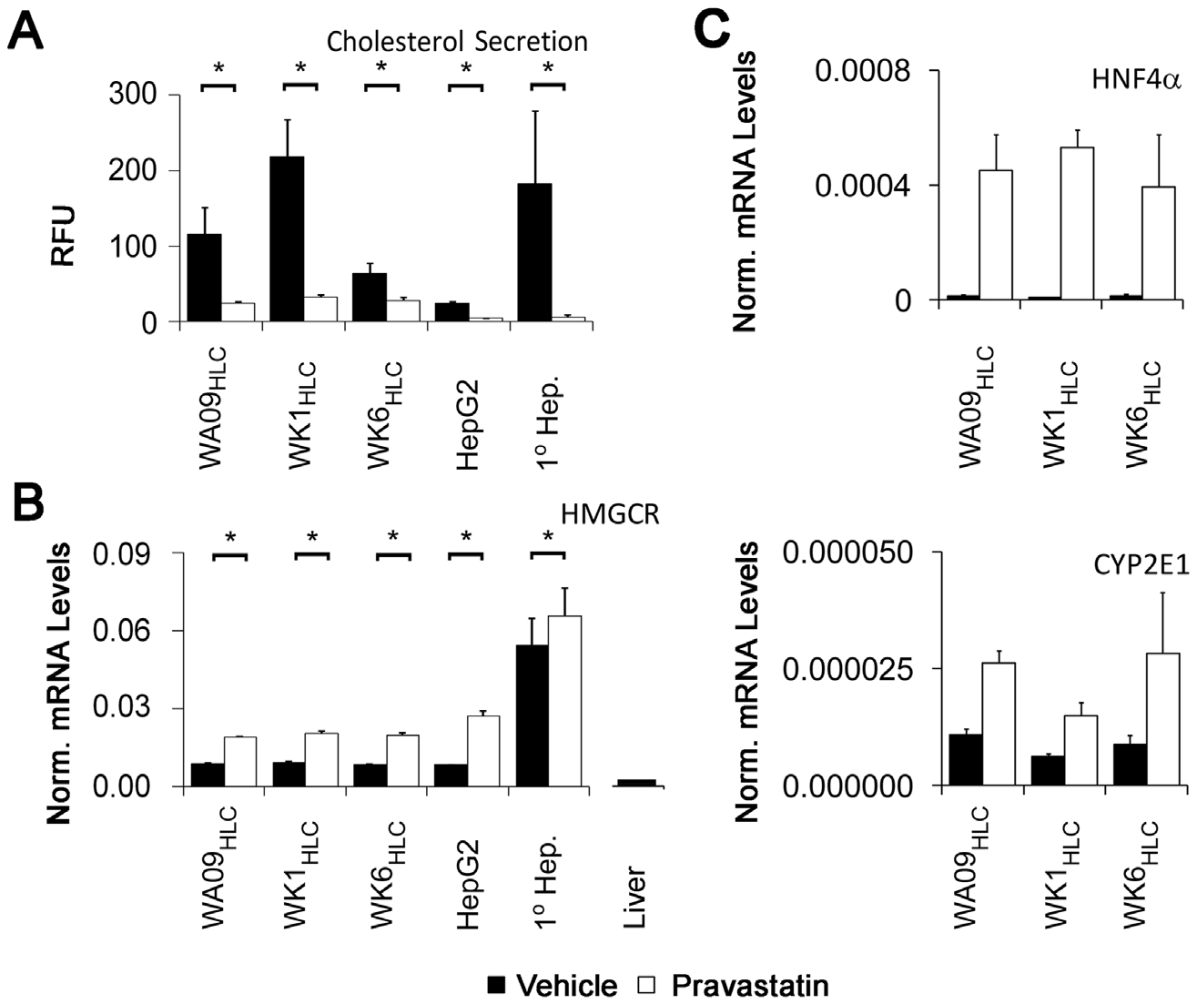
Pravastatin induced reduction in cholesterol secretion of stage 3B HLCs derived from iPSCs. (A) Suppression of cholesterol secretion by treatment of HLCs with pravastatin at 10 μM for 48 hours, as assayed by quantitative fluorometric assessment for cholesterol in cell-fee conditioned medium. T-tests on triplicate values from treated and untreated cells were used to generate p-values. (B) Analysis of HMGCR transcriptional regulation by pravastatin (for cell-type nomenclature see Fig. 3A legend). Also shown is the HMGCR mRNA level from a 2 year old female liver sample. Scales for the liver and HLC samples are identical. (C) Analysis of HNF4α and CYP2E1 transcriptional regulation by pravastatin (for cell-type nomenclature see Fig. 3A legend). Error bars represent the standard error of the mean.

Circulating endogenously synthesized cholesterol is exclusively of hepatocyte origin [34] and its secretion in the form of soluble lipoprotein particles is a hallmark of periportal hepatocytes. Strikingly, conditioned medium from stage 3B HLCs derived from all three pluripotent cells contained significant amounts of soluble cholesterol (Fig. 6A). In contrast, fibroblast lines hDF1 and hDF6 failed to secrete detectable cholesterol into cell culture medium (data not shown). Remarkably, amounts of cholesterol secreted by our HLCs was comparable to amounts secreted by primary hepatocytes and exceeded levels secreted by HepG2 cells between twofold for HLCs derived from the WK6_iPSC_ line to ten-fold for HLCs derived from the WK1_iPSC_ line (Fig. 6A). Moreover, all HLCs treated with the HMG-CoA reductase inhibitor pravastatin showed robust reduction in cholesterol secretion ranging from more than 50% for HLCs derived from the WK6_iPSC_ line to nearly 90% for HLCs derived from the WK1_iPSC_ line and 85% for HLCs derived from WA09 ESCs (Fig. 6A). This reduction was statistically significant with p-values of less than 0.01 in each case and mirrored the reduction found in both HepG2 cells and primary hepatocytes (Fig. 6A). Importantly, HMGCR mRNA expression was observed in all stage 3B HLCs at high levels and similar to the one found in HepG2 cells. While they exceeded the amount observed in human liver by 3- to 5-fold (Fig. 6B), an almost nine-fold higher levels of HMGCR were detected in primary hepatocytes (Fig. 6B). Statin treatment is known to affect expression of genes involved in cholesterol metabolism due to autoregulatory transcriptional mechanisms. Treatment of stage 3B HLCs with pravastatin induced statistically significant and robust up-regulation of HMGCR mRNA with p-values of less than 0.01 (Fig. 6B), consistent with previous observations in the mouse model [35] and our own observations for HepG2 cells and primary hepatocytes (Fig. 6B). Finally, HNF4αand CYP2E1 mRNA were induced more than 40-fold and 2- to 3-fold, respectively, upon pravastatin treatment of HLCs derived from all three pluripotent cell lines used in this study (Fig. 6C).

## Discussion

Understanding the idiosyncratic variations in the mechanisms controlling drug metabolism is of critical importance for drug dosing and the estimation of toxicological variation during drug development. Therefore, a major focus has been placed in most prior studies on the expression of proteins participating in the phase 1 and phase 2 detoxification systems at levels comparable to nascent primary hepatocytes. Among these, CYP proteins have been at the center of attention since their function accounts for almost three quarters of all activities required for the detoxification of drugs [36]. CYP levels vary substantially across individuals and may be attributed to gene deletions, gene amplifications, promoter polymorphisms and ORF polymorphisms [37]. For example, CYP2D6 is deleted in 20–30 million subjects and amplified in another 15–20 million europeans. Moreover, single nucleotide polymorphisms further affect function and expression levels of these proteins. A wide variety of tools including purified CYPs, transgenic expression of selected CYPs in cell lines, humanized mice or neutralizing antibodies have been developed to determine the specific CYP that metabolizes a particular drug. The effect of polymorphisms that can be studied with these tools is limited to sequence variations occurring in the coding region; the impact of single nucleotide changes outside of the open reading frame or gene amplifications cannot be investigated via this route. However, hepatic differentiation of donor specific iPSCs provides a valuable resource that can capture the effect of donor specific CYP gene variants on the metabolism of selected drugs or toxicants. Therefore, prior studies of HLCs derived from pluripotent cells have focused on the production of cells that express CYP proteins [18], [38], [39] and produced cells with features of perivenular hepatocytes. Common to HLCs produced in these studies are the generally low levels of CYP proteins which could be attributed to similar issues affecting primary hepatocytes cultured for more than only a few days.

We have differentiated both ES and iPS cells into HLCs and we observed varied up-regulation of HLC markers ranging from 40 fold for APOA2, to up to 1600 fold for APOA1, to two orders of magnitude for ALB and five orders magnitude for the fetal hepatocyte marker AFP. Thus, up-regulation of HLC markers was generally in the range observed in many other studies that reported HLC marker expression in relation to pluripotent stem cell derived baselines [40], [41], [42], [43], [44], [45]. However, when standardized to established in vitro models including HepG2 cells or expression in liver, our data clearly show that the current protocol does not yet yield comparable HLCs. We therefore propose that novel HLC differentiation procedures should be evaluated on the basis of comparison to all HepG2 cells, primary hepatocytes and post natal liver.

HLC marker expression was generally higher in HLCs derived from ESCs but the differences remain mostly within an order of magnitude and reflect differing efficiencies of hepatocyte differentiation. Our protocol started with endoderm induction of pluripotent stem cell colonies precluding the determination of precise cell numbers used in each experiment and, based on the rates of cell death during endoderm induction, one likely cause for inefficient differentiation includes insufficient numbers of starting cells. It is also possible that some partial differentiation of the starting cells further impeded the differentiation process but WA09_HLCs_ expressed higher levels of HLC markers than HLCs derived from iPSCs that expressed much higher levels of the pluripotency marker SSEA4 than our WA09 cells. We therefore conclude that the starting cell density had the greatest impact on differentiation efficiencies although we cannot rule that epigenetic memory persistent in our iPSCs also affected the process.

Together, our data suggest that the HLCs produced by even the latest methodologies show a much greater resemblance to embryonic forms of periportal-like rather than perivenular-like HLCs. We offer three major lines of evidence to support this conclusion: (1) Our HLCs secrete cholesterol and express high levels of HMGCR (Fig. 5B), two clinically-relevant hepatocyte functions that, to our knowledge, are the most prominent features of periportal hepatocytes. (2) Our cells readily express several markers of cholesterol homeostasis including APOA1, APOA2, APOC3, and LDLR at both mRNA and protein level (Fig. 4), as well as APOE mRNA which encodes a lipoprotein that is enriched in male periportal hepatocytes [14]. (3) Our HLCs respond to statin treatment with reduced cholesterol secretion and transcriptional responses characteristic of statin treatment (Fig. 6). While the up-regulation of HMG-CoA reductase is consistent with results from previous studies using freshly prepared primary hepatocytes [46] the up-regulation of HNF4αand CYP2E1 transcription was unexpected since HNF4αup-regulation had only been described for treatment of hepatocytes with atorvastatin [47], and a previous study reported that pravastatin does not facilitate the up-regulation of the CYP class of HNF4α target genes [48].

Periportal HLCs from human iPS cells provide a powerful new platform for the study of hepatic functions that impact cardiovascular health as mediated by serum cholesterol. It is well established that human genetic variation impacts cholesterol homeostasis. Extreme examples of genetic effects include familial hypercholesterolemias, often caused by defects in the LDLR gene [49], and dyslipidemias arising from mutations in genes encoding APOA4, B, C2, C3, and E [50], PCSK9 [51], and CYP7A1 [52]. These are examples of monogenic influences on cholesterol homeostasis. However, clinical findings suggest that overtly normal individuals may each have a unique, and likely polygenic, set of genetic influences that collude to set baseline serum cholesterol levels, thus defining an intrinsic “cholesterol thermostat” whereby serum cholesterol levels are maintained. In support of this hypothesis and assuming similar degrees of differentiation, we found that HLC derived from three independent pluripotent cell lines (each harboring a complete and unique human genome) expressed similar amounts of HMGCR transcripts (Fig.5B), yet cholesterol secretion and statin responses varied considerably between these cells. These differing magnitudes of cholesterol secretion and statin response could be caused by genomic variation that differs between each of these cell lines which impacts cholesterol metabolism. Alternate explanations for this variation include non-specific cholesterol release due to cell death, different degrees of hepatic maturity in the differentiated cultures and the generation of HLC subpopulations that represent cells with characteristics specific to hepatocytes from the different metabolic zones encountered in the liver [11], [12]. However, we did not observe differing levels of cell death (not shown) and our HLCs reduce cholesterol release dramatically in the presence of pravastatin, a drug not known for inhibition of cell death. At present, due to the absence of appropriate markers, we cannot exclude the possibility that variations in hepatic maturity or in the number of zonally distinct subpopulations composing the final HLC culture contributed to the observed differences in cholesterol secretion. Comparison of HLCs derived from further donors and genome-wide association of identifiable polymorphisms with variations in cholesterol metabolism in future studies will greatly aid in answering this open question. Such studies are possible only using iPSCs since only these cells can be derived from a sample of subjects large enough to capture the effect of genetic variation on mechanisms for cholesterol homeostasis. Moreover, only HLCs derived from iPSCs are amenable to genetic manipulation for rescuing of genetically impaired cholesterol homeostasis. In this context, HLCs derived from iPS cells are superior to the current gold standard of HepG2 cells, primary hepatocytes or liver tissue.

Lastly, our iPS cell culture model removes the confounding variable of dietary cholesterol, which is difficult to control in studies of human subjects. Our cholesterogenic HLC model thus provides a novel and effective way to examine the contribution of individualized genomic variation to cholesterol homeostasis, with obvious implications for advances in cardiovascular health research.

## Supporting Information

Figure S1 **Morphological assessment of pluripotent stem cell lines H9, WK1 and WK6 by light microscopy.** Phase contrast microscopy of pluripotent stem cell lines WA09, WK1 and WK6 grown on feeder cells with human embryonic stem cell medium (see materials and methods) (A) and on matrigel with mTESR1 (Stem Cell Technologies) (B). Both WK1 and WK6 cells show the typical colony morphology with distinct edges independent of the growth conditions on feeder cells or matrigel as was observed for WA09 embryonic stem cells. Individual iPSCs also show the large ratio of nuclear to total cell volume typical for human embryonic stem cells (WA09).
(TIF)

Figure S2 **Directed differentiation of hiPSC line WK1 to HLCs.** Immunofluorescent detection of the hepatic lineage markers SOX17 and ALB during hepatic differentiation of hiPSC line WK1. The normal human dermal fibroblast line hDF1 (row 1) was reprogrammed to yield hiPSC line WK1 (row 2), WK1, was subjected to the three-stage directed differentiation procedure outlined above (Fig.1a). Undifferentiated WK1 cells, parental hDFs and cells at successive stages of hepatic differentiation were assessed by immunofluorescence to detect definitive endoderm marker SOX17, and the definitive hepatocyte marker ALB. Note the progression from SOX17 positive to albumin positive cells over the course of differentiation. All images are of cell cultures grown in plastic tissue-culture wells, which were fixed in situ and subjected to immunofluorescence, then imaged by inverted fluorescence microscopy.
(TIF)

Table S1 **List of qRT- and RT-PCR primer sequences used in this study.**
(DOCX)

Table S2 **Regulation of gene expression for selected genes during hepatic differentiation of WA09 hES cells.**
(DOCX)

Table S3 **Regulation of gene expression for selected genes during hepatic differentiation of WK1 iPS cells derived from hDF1 fibroblasts.**
(DOCX)

Table S4 **Regulation of gene expression for selected genes during hepatic differentiation of WK6 iPS cells derived from hDF6 fibroblasts.**
(DOCX)
