# Supplementary figures and images for: Cholesterol-Secreting and Statin-Responsive Hepatocytes from Human ES and iPS Cells to Model Hepatic Involvement in Cardiovascular Health
Source: PLoS One. 2013 Jul 11;8(7):e67296. doi: 10.1371/journal.pone.0067296 (PMC3708950; doi:10.1371/journal.pone.0067296)

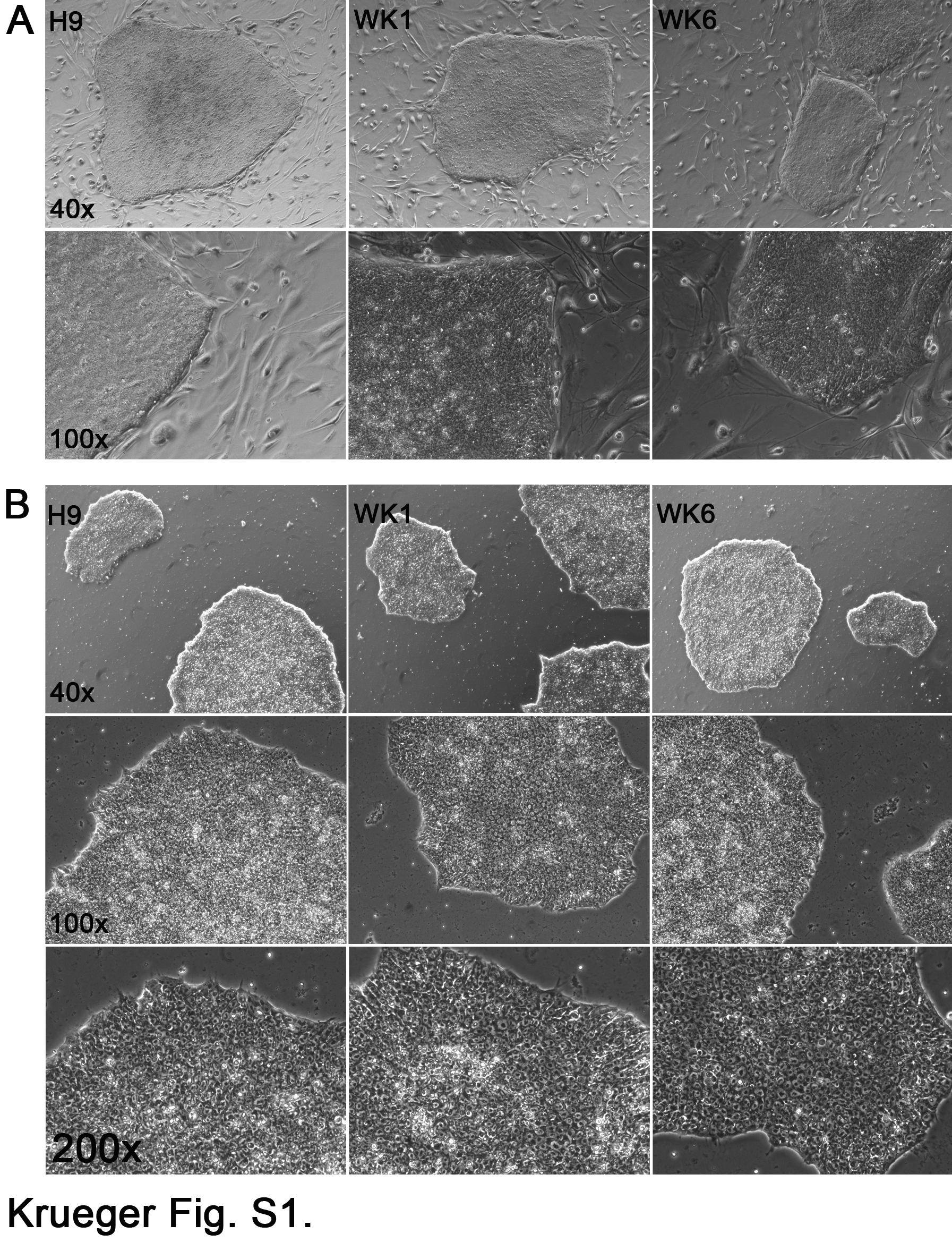

Supplement: Figure S1 — Morphological assessment of pluripotent stem cell lines H9, WK1 and WK6 by light microscopy. Phase contrast microscopy of pluripotent stem cell lines WA09, WK1 and WK6 grown on feeder cells with human embryonic stem cell medium (see materials and methods) (A) and on matrigel with mTESR1 (Stem Cell Technologies) (B). Both WK1 and WK6 cells show the typical colony morphology with distinct edges independent of the growth conditions on feeder cells or matrigel as was observed for WA09 embryonic stem cells. Individual iPSCs also show the large ratio of nuclear to total cell volume typical for human embryonic stem cells (WA09). (TIF) [file pone.0067296.s001.tif]

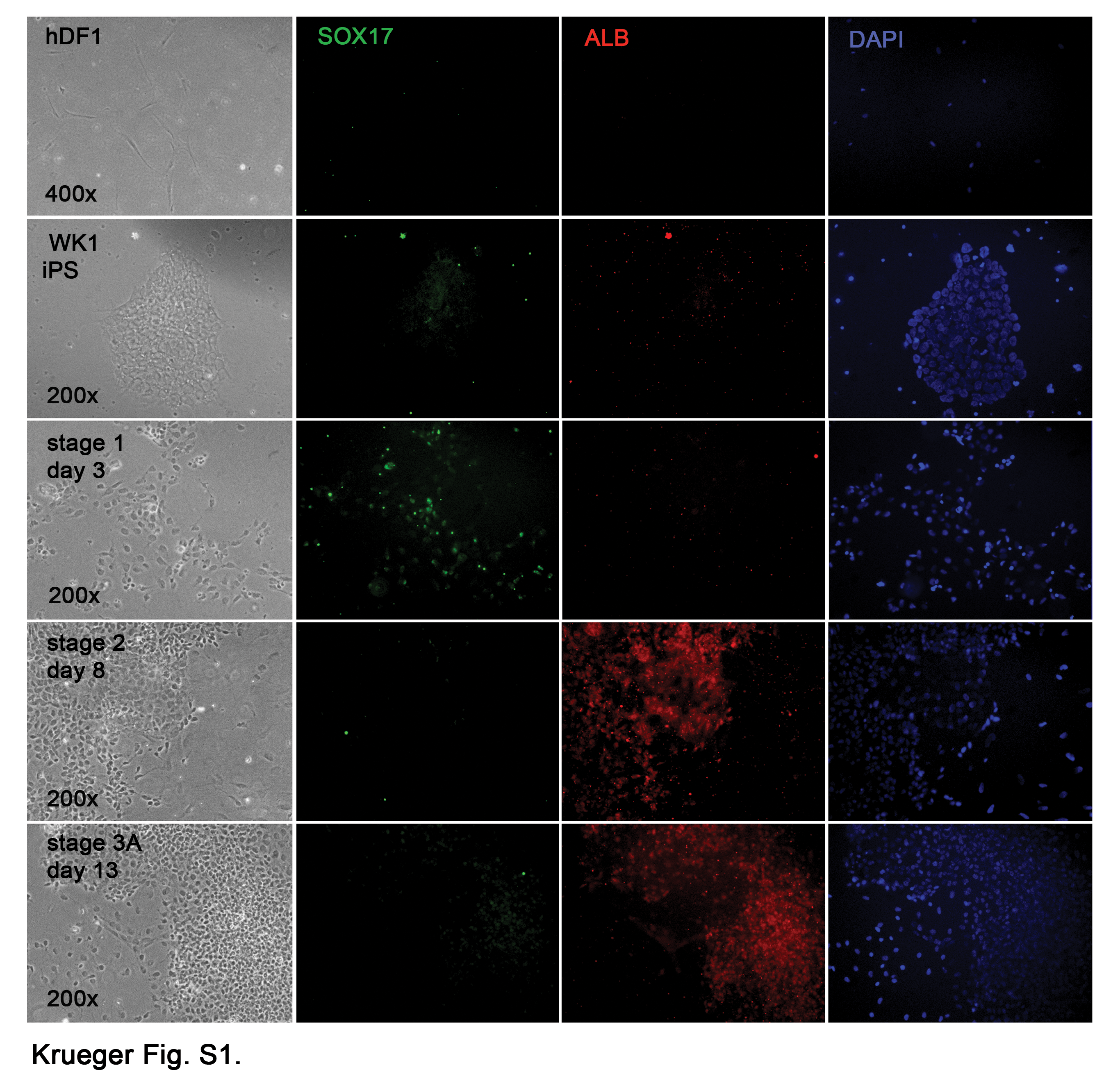

Supplement: Figure S2 — Directed differentiation of hiPSC line WK1 to HLCs. Immunofluorescent detection of the hepatic lineage markers SOX17 and ALB during hepatic differentiation of hiPSC line WK1. The normal human dermal fibroblast line hDF1 (row 1) was reprogrammed to yield hiPSC line WK1 (row 2), WK1, was subjected to the three-stage directed differentiation procedure outlined above (Fig.1a). Undifferentiated WK1 cells, parental hDFs and cells at successive stages of hepatic differentiation were assessed by immunofluorescence to detect definitive endoderm marker SOX17, and the definitive hepatocyte marker ALB. Note the progression from SOX17 positive to albumin positive cells over the course of differentiation. All images are of cell cultures grown in plastic tissue-culture wells, which were fixed in situ and subjected to immunofluorescence, then imaged by inverted fluorescence microscopy. (TIF) [file pone.0067296.s002.tif]
