# Supplementary material for: Cholesterol-Secreting and Statin-Responsive Hepatocytes from Human ES and iPS Cells to Model Hepatic Involvement in Cardiovascular Health
Source: PLoS One. 2013 Jul 11;8(7):e67296. doi: 10.1371/journal.pone.0067296 (PMC3708950; doi:10.1371/journal.pone.0067296)
Supplement: Table S1 — List of qRT- and RT-PCR primer sequences used in this study. (DOCX) [file pone.0067296.s003.docx]

| **Table S1: List of qRT- and RT-PCR primer sequences used in this study** | | |
| --- | --- | --- |
| Gene | Sequence of forward primer | Sequence of reverse primer |
| ACTB | 5’-GGCACCCAGCACAATGAAG-3’ | 5’-GCCGATCCACACGGAGTACT-3’ |
| AFP | 5’-CCCACTGGAGATGAACAGTCTTC-3’ | 5’-TGGCAAAGTTCTTCCAGAAAGG-3’ |
| ALB | 5’-TGCTGAGGCAAAGGATGTCTT-3’ | 5’-TCAGGATGCCTTCTTGCATATTC-3’ |
| APOA1 | 5’-ACAGCGGCAGAGACTATGTG-3’ | 5’-CCCAGTTGTCAAGGAGCTTTAG-3’ |
| APOA2 | 5’-ACCGTGACTGACTATGGCAAG-3’ | 5’-TCTTGATCAGGGGTGTCAGC-3’ |
| APOA4 | 5’-AGAAATCTGAACTCACCCAGCAA-3’ | 5’-GGTCACCTGCGTAAGTGTTCAC-3’ |
| APOA5 | 5’-GGAGCAGATCCATCAGCAGAA-3’ | 5’-AGGTCTTGCTCAAGGCTGTCTTT-3’ |
| APOB | 5’-GGAGTTTGCTGCAGCCATGT-3’ | 5’-CCTGCTTCCCTTCTGGAATG-3’ |
| APOC1 | 5’-AACAGAGTGAACTTTCTGCCAAGA-3’ | 5’-GTTTCTCCTTCACTTTCTGAAATGTCT-3’ |
| APOC2 | 5’-ATACCTGCCCGCTGTAGATGA-3’ | 5’-TGGCTGCTGTGCTTTTGC-3’ |
| APOC3 | 5’-TGTCTGCTCAGTTCATCCCTAGAG-3’ | 5’-GGAGGGCAACAACAAGGAGTAC-3’ |
| APOC4 | 5’-AGAAATGTCCCTCCTCAGAAACAG-3’ | 5’-TCCAGCGACTCATCTTTAGCTTT-3’ |
| APOD | 5’-TCCGGTGCAGGAGAATTTTG-3’ | 5’-GGGATCTTCTCAATTTCGTACCA-3’ |
| APOE | 5’-GCGTTGCTGGTCACATTCCT-3’ | 5’-TCTGTCTCCACCGCTTGCT-3’ |
| APOF | 5’-AAACCTACCAAGGCAGTCTCACTT-3’ | 5’-ACGCATGTCTGGAGCAGAGTAC-3’ |
| APOH | 5’-ACAGGACTGTGGCCCATCA-3’ | 5’-TAAGATTCCAGCAAAAGGACATACTC-3’ |
| APOL1 | 5’-GGCCTGGAACGGATTCGT-3’ | 5’-ACGGAGCTCATCTGCCTCAT-3’ |
| APOL2 | 5’-TGAAGCCTGGAATGGATTCG-3’ | 5’-ACGGAGCTCATCTGCCTCAT-3’ |
| APOL3 | 5’-AGCCTGGAAGAGATTCGTGACT-3’ | 5’-TCGTAGAGAGCATCTGCCTCATC-3’ |
| APOL4 | 5’-TGAAGCCTGGAAGAGATTTGTG-3’ | 5’-TTCAGAGCTTCATAGAGAGCATCTG-3’ |
| APOL6 | 5’-AGGCTGGTGTTGGTTTGCA-3’ | 5’-GTAGCTCCACGTCTTCACACAGA-3’ |
| APOM | 5’-CCGATGCAGCTCCACCTT-3’ | 5’-CAGTCAGGTGGTAGATCCATTTCC-3’ |
| APOO | 5’-ACACTATTGCGAGCCATACACAA-3’ | 5’-CATCTTGGGCTTAGTTTGGGAGTA-3’ |
| CYP2E1 | 5’-GGGCTCCTGATTCTCATGAAATA-3’ | 5’-CACCCTGTCAATTTCTTCATGGA-3’ |
| GATA6 | 5’-CCTCATCAAGCCGCAGAAG-3’ | 5’-ACAGTTGGCACAGGACAATCC-3’ |
| GHR | 5’-ACTAGCAATGGTGGTACAGTGG-3’ | 5’-AAGTCCAGTTGAGGGCAATG-3’ |
| GSTA1 | 5’-CATTGCCAGCAAATACAACC-3’ | 5’-GGCATCTTTTTCCTCAGGTG-3’ |
| HMGCR | 5’-CAAAGTTTGCCCTCAGTTCCA-3’ | 5’-GCCATTCCACGAGCAATATTTT-3’ |
| HNF4A | 5’-CCAAGAGATCCATGGTGTTCAA-3’ | 5’-GCCGAGGGACAATGTAGTCATT-3’ |
| IGF1 | 5’-TCTTCAGTTCGTGTGTGGAGACA-3’ | 5’-TCCGACTGCTGGAGCCATAC-3’ |
| IGF2 | 5’-CTGGTGGACACCCTCCAGTTC-3’ | 5’-GCGGAAACAGCACTCCTCAAC-3’ |
| IGFBP2 | 5’-GAGGGCACTTGTGAGAAGC-3’ | 5’-ACATGTTCATGGTGCTGTCC-3’ |
| LDLR | 5’-CTGGTCAGATGAACCCATCAAA-3’ | 5’-GCCGTTGTTGTCCAAGCATT-3’ |
| MDR3 | 5’-CTTCAGCCGGCATTTTCAG-3’ | 5’-CATCATCGCCTGGTCCAAA-3’ |
| POU5F1 | 5’-CCCCTGGTGCCGTGAAG-3’ | 5’-CTCGAGTTCTTTCTGCAGAGCTT-3’ |
| ZFP42 | 5’-GCCTTCACTCTAGTAGTGCTCACAGT-3’ | 5’-GGCAGTAGTGATCTGAGTAAGCTGTCT-3’ |
| RXRA | 5’-GCACATCTGCGCCATCTG-3’ | 5’-TCGCAGCTGTACACTCCATAGTG-3’ |
| SCARB1 | 5’-CTCCTTGTTCCTGGACATCCA-3’ | 5’-TGCAGTTTCACAGAGCAGTTCA-3’ |
| VIM  NES  NETO2  SOX17  AFP  KDR  MANTN1  T  MYF5  GAPDH | 5’-CCGAAAACACCCTGCAATCT-3’  5’-GAAGGGCAATCACAACAGGTG-3’  5’-TCAGGGATTGTCTTGGTCCT-3’  5’-GTGGACCGCACGGAATTTG-3’  5’-TGCAATTGAGAAACCCACTG-3’  5’-GCCATGTGGTCTCTCTGGTT-3’  5’-CATCCAGTTCGCTATCACCA-3’  5’-TATGAGCCTCGAATCCACATAGT-3’  5’-CTGCCAGTTCTCACCTTCTGA-3’  5’-ACCACAGTCCATGCCATCAC-3’ | 5’-AAGGTCAAGACGTGCCAGAGA-3’  5’-GGGGCCACATCATCTTCCA-3’  5’-TCCATGGAACTGAGGTTGGT-3’  5’-GGAGATTCACACCGGAGTCA-3’  5’-CAGCTTGTGACAGGTTCTGG-3’  5’-TTCCTCCAACTGCCAATACC-3’  5’-TTCCTGGACAGCTTCTCGAT-3’  5’-CCTCGTTCTGATAAGCAGTCAC-3’  5’-AACTCGTCCCCAAATTCACCC-3’  5’-TCCACCACCCTGTTGCTGTA-3’ |

| **Table S2A. Regulation of gene expression for selected genes during hepatic differentiation of WA09 hES cells.** | | | | | |
| --- | --- | --- | --- | --- | --- |
| a | b | c | d | e | f |
| AFP | 2.69 | 2.69 | 276937.74 | 144711.57 | 97.71 |
| ALB | 3.89 | 3.89 | 1638.82 | 917.18 | 19056629.82 |
| CYP2E1 | 3.24 | 3.24 | 109.9 | 11.09 | 63554752.01 |
| GATA6 | 15.48 | 2.49 | 2338.67 | 1155.35 | 11405.93 |
| GHR | 432.5 | 80.64 | 4810.14 | 1658.55 | 203757.93 |
| GSTA1 | 111.81 | 19.75 | 6112.95 | 1741.85 | 13076713.49 |
| HMGCR | 139673.64 | 2698.58 | 112107.18 | 3428.44 | 26680.47 |
| HNF4A | ND | ND | 136.46 | 44.52 | 450602.77 |
| IGF1 | ND | ND | 654.06 | 176.66 | 10914.11 |
| IGF2 | 109.84 | 21.38 | 1186250.87 | 601011.5 | 283796.38 |
| IGFBP2 | 204369.19 | 10817.37 | 1523211.44 | 244744.87 | 677798.38 |
| LDLR | 12262.51 | 666.34 | 9035.49 | 838.04 | 79711.3 |
| MDR3 | 168.24 | 23.3 | 1720.04 | 376.19 | 143879.02 |
| POU5F1 | 1292187.21 | 95038.83 | 47711.13 | 11803.83 | 44777.82 |
| ZFP42 | 38229.82 | 3562.91 | 3499.82 | 948.12 | ND |
| RXRA | 3300.48 | 270.33 | 6165.2 | 707.62 | 432546.79 |
| SCARB1 | 9460.66 | 698.75 | 15325.13 | 3950.78 | 163337.52 |
| VIM | 116072.98 | 12986.62 | 1997610.61 | 52500.56 | 231796.08 |
| APOA1 | 147.22 | 41.36 | 238649 | 86977.16 | 17912913.4 |
| APOA2 | 2555.1 | 199.43 | 126082.28 | 46441.9 | 37243792.79 |
| APOA4 | ND | ND | 22729.71 | 12062.87 | 15842.33 |
| APOA5 | ND | ND | ND | ND | 129671.03 |
| APOB | 43.22 | 4.01 | 2592.94 | 1257.73 | 1083671.95 |
| APOC1 | 23794.2 | 1485.76 | 14748.36 | 4665.34 | 6341957 |
| APOC2 | 29.26 | 3.72 | 3370.79 | 1526.51 | 1876348.87 |
| APOC3 | 1.8 | 1.8 | 2769.99 | 1468.73 | 10224285.31 |
| APOC4 | ND | ND | ND | ND | 207895.16 |
| APOD | ND | ND | 246.65 | 116.83 | 554.67 |
| APOE | 186003.62 | 12136.61 | 157104.67 | 40114.65 | 6466244.66 |
| APOF | ND | ND | ND | ND | 128240.88 |
| APOH | 0.59 | 0.59 | 38.83 | 20.07 | 2326124.14 |
| APOL1 | 1213.11 | 77.25 | 1319.28 | 288.95 | 66427.67 |
| APOL2 | 6123.34 | 278.15 | 5697.81 | 1027.94 | 48058.26 |
| APOL3 | 167.56 | 89.44 | 143.06 | 23.79 | 9806.32 |
| APOL4 | 46.66 | 3.12 | 86.32 | 49.65 | 17712.7 |
| APOL6 | 111.72 | 21.05 | 625.08 | 128.34 | 34456.69 |
| APOM | 2491.06 | 109.47 | 9241.77 | 1623.9 | 119652.97 |
| APOO | 17978.76 | 972.84 | 15572.37 | 1019.38 | 5539.33 |
| Values for mRNAs analyzed in this study are given as fold β-actin mRNA amounts multiplied by 10^-7^.  Abbreviations: a: mRNA; b: WA09-ESCs Mean; c: WA09-ESCs SEM; d: WA09-HLCs Mean; e: WA09-HLCs SEM; f: Liver; SEM – Standard error of the mean | | | | | |

| **Table S2B. Regulation of gene expression for selected genes during hepatic differentiation of WK1 iPS cells derived from hDF1 fibroblasts.** | | | | | | | |
| --- | --- | --- | --- | --- | --- | --- | --- |
| a | b | c | d | e | f | g | h |
| AFP | 21.74 | 21.74 | 2.62 | 2.62 | 34472.47 | 8358.59 | 97.71 |
| ALB | 2.02 | 2.02 | 3.51 | 0.34 | 270.84 | 63.79 | 19056629.82 |
| CYP2E1 | 324.97 | 108.41 | 3.01 | 1.63 | 61.59 | 5.11 | 63554752.0 |
| GATA6 | 145.65 | 58.94 | 205.03 | 36.87 | 1156.21 | 98.01 | 11405.93 |
| GHR | 1141.17 | 86.47 | 303.05 | 43.99 | 1225.99 | 211.08 | 203757.93 |
| GSTA1 | ND | ND | 104.7 | 21.36 | 3532.47 | 2245.97 | 13076713.5 |
| HMGCR | 10850.84 | 60.17 | 106255.83 | 10205.9 | 79843.26 | 3457.42 | 26680.47 |
| HNF4A | ND | ND | 1.32 | 0.67 | 83.54 | 3.92 | 450602.77 |
| IGF1 | 13.7 | 2.8 | 2.78 | 1.68 | 387.63 | 62.74 | 10914.11 |
| IGF2 | 40114.98 | 5348.04 | 137.94 | 31.71 | 243868.77 | 27382.84 | 283796.38 |
| IGFBP2 | 8526.74 | 909.13 | 160759.29 | 4936.51 | 577315.66 | 50921.05 | 677798.38 |
| LDLR | 7910.43 | 2192.67 | 12674.67 | 1480.25 | 8016.96 | 123.48 | 79711.3 |
| MDR3 | 77.48 | 11.53 | 244.05 | 16.6 | 1152.18 | 235.5 | 143879.02 |
| POU5F1 | 6247.34 | 715.68 | 1291597.71 | 85735.7 | 32267.05 | 8029.92 | 44777.82 |
| ZFP42 | ND | ND | 29422.44 | 2782.58 | 3169.01 | 164.63 | ND |
| RXRA | 5071.62 | 1596.88 | 3007.77 | 102.02 | 5113.22 | 255.3 | 432546.79 |
| SCARB1 | 4607.75 | 1157.85 | 11467.42 | 913.62 | 5677.23 | 475.86 | 163337.52 |
| VIM | 3546637.96 | 584635.68 | 109323.04 | 6998.24 | 1551061.58 | 126512.34 | 231796.08 |
| APOA1 | 16.42 | 8.63 | 2018.13 | 435.18 | 122555.18 | 16160.27 | 17912913.4 |
| APOA2 | 206.16 | 44.48 | 1214.48 | 318.76 | 46905.69 | 4549.63 | 37243792.79 |
| APOA4 | ND | ND | 11.58 | 5.85 | 3271.67 | 616.06 | 15842.33 |
| APOA5 | ND | ND | ND | ND | ND | ND | 129671.03 |
| APOB | 1.71 | 1.71 | 36.72 | 3.05 | 260.58 | 20.94 | 1083671.95 |
| APOC1 | 33 | 17.25 | 17546.97 | 2339.46 | 5202.67 | 643.88 | 6341957 |
| APOC2 | ND | ND | 18.41 | 3.63 | 392.6 | 58.21 | 1876348.87 |
| APOC3 | ND | ND | 2.48 | 2.48 | 982.73 | 312.81 | 10224285.31 |
| APOC4 | ND | ND | ND | ND | ND | ND | 207895.16 |
| APOD | 37586.09 | 13095.06 | 5.9 | 5.9 | 1408.64 | 208.45 | 554.67 |
| APOE | 200.97 | 81.12 | 154261.43 | 5059.78 | 53544.93 | 3728.84 | 6466244.66 |
| APOF | ND | ND | ND | ND | ND | ND | 128240.88 |
| APOH | ND | ND | 1.53 | 1.53 | 36.88 | 12.6 | 2326124.14 |
| APOL1 | 13115.95 | 553.06 | 909.76 | 112.86 | 1156.5 | 88.32 | 66427.67 |
| APOL2 | 23108.42 | 698.85 | 4190.27 | 210.65 | 5484.82 | 418.81 | 48058.26 |
| APOL3 | 3099.14 | 32.08 | 92.14 | 3.94 | 127.98 | 12.55 | 9806.32 |
| APOL4 | 3637.02 | 80.17 | 31 | 5.11 | 178.4 | 20.94 | 17712.7 |
| APOL6 | 30737.92 | 764.98 | 59.33 | 12.96 | 689.55 | 72.39 | 34456.69 |
| APOM | 2168.95 | 291.62 | 2126.47 | 125.06 | 4605.9 | 598.38 | 119652.97 |
| APOO | 2996.82 | 264.18 | 19543.25 | 1088.22 | 32371.5 | 2604.93 | 5539.33 |
| Values for mRNAs analyzed in this study are given as fold β-actin mRNA amounts multiplied by 10^-7^.Abbreviations: a: mRNA; b: hDF1 Mean; c: hDF1 SEM; d: WK1-iPSCs Mean; e: WK1-iPSCs SEM; f: WK1-HLCs Mean; g: WK1-HLCs SEM; h: Liver; SEM – Standard error of the mean | | | | | | | |

| **Table S2C. Regulation of gene expression for selected genes during hepatic differentiation of WK6 iPS cells derived from hDF6 fibroblasts.** | | | | | | | |
| --- | --- | --- | --- | --- | --- | --- | --- |
| a | b | c | d | e | f | g | h |
| AFP | ND | ND | 1.17 | 0.59 | 85011.88 | 36140.18 | 97.71 |
| ALB | 2.9 | 2.9 | 2.53 | 0.63 | 378.75 | 148.07 | 19056629.82 |
| CYP2E1 | 466.35 | 8.31 | ND | ND | 87 | 21.14 | 63554752.01 |
| GATA6 | 17867.38 | 587.71 | 917.71 | 90.85 | 1649.32 | 646.18 | 11405.93 |
| GHR | 2374.43 | 313.02 | 371.78 | 23.9 | 3132.84 | 468 | 203757.93 |
| GSTA1 | ND | ND | 296.38 | 14.72 | 2852.21 | 1282.2 | 13076713.49 |
| HMGCR | 13477.82 | 561.54 | 79246.08 | 3119.68 | 60179.92 | 644.71 | 26680.47 |
| HNF4A | ND | ND | 4.8 | 0.33 | 134.28 | 58 | 450602.77 |
| IGF1 | 1836.89 | 424.55 | 0.63 | 0.63 | 702.06 | 113.64 | 10914.11 |
| IGF2 | 257.78 | 74.14 | 114.15 | 10.04 | 563589.38 | 47333.45 | 283796.38 |
| IGFBP2 | 163.84 | 37.39 | 132473.09 | 1468.45 | 429994.94 | 18682.21 | 677798.38 |
| LDLR | 11451.43 | 1259.32 | 10001.46 | 317.49 | 8016.22 | 923.3 | 79711.3 |
| MDR3 | 379.49 | 57.69 | 148.76 | 36.87 | 829.99 | 173.78 | 143879.02 |
| POU5F1 | 6841.93 | 396.93 | 1175284.2 | 79935.15 | 24395.33 | 2060.62 | 44777.82 |
| ZFP42 | ND | ND | 29741.49 | 2266.31 | 4052.92 | 289.1 | ND |
| RXRA | 7427.21 | 341.37 | 2574.29 | 124.02 | 4676.36 | 71.36 | 432546.79 |
| SCARB1 | 3397.17 | 136.48 | 8287.23 | 476.94 | 5606.01 | 460.81 | 163337.52 |
| VIM | 2269342.39 | 215472.43 | 94132.81 | 7569.89 | 1080069.59 | 114732.62 | 231796.08 |
| APOA1 | 2.16 | 2.16 | 383.93 | 54.61 | 118772.7 | 53018.68 | 17912913.4 |
| APOA2 | 217.62 | 46.11 | 2381.94 | 237.82 | 66089.52 | 27530.87 | 37243792.79 |
| APOA4 | ND | ND | ND | ND | 5535.89 | 2736.64 | 15842.33 |
| APOA5 | ND | ND | ND | ND | ND | ND | 129671.03 |
| APOB | ND | ND | 29.14 | 8.31 | 649.72 | 255.01 | 1083671.95 |
| APOC1 | 174 | 20.52 | 19840.2 | 1711.31 | 7152.69 | 1706.58 | 6341957 |
| APOC2 | ND | ND | 8.74 | 4.17 | 981.64 | 405.41 | 1876348.87 |
| APOC3 | ND | ND | ND | ND | 985.85 | 404.2 | 10224285.31 |
| APOC4 | ND | ND | ND | ND | ND | ND | 207895.16 |
| APOD | 14373.43 | 1040.89 | 6.09 | 3.05 | 972.6 | 406.46 | 554.67 |
| APOE | 1928.59 | 46.58 | 146907.45 | 7906.73 | 88533.68 | 5275.19 | 6466244.66 |
| APOF | ND | ND | ND | ND | ND | ND | 128240.88 |
| APOH | ND | ND | 2.17 | 2.17 | 28.48 | 14.49 | 2326124.14 |
| APOL1 | 9607.99 | 467.25 | 941.44 | 150.74 | 1334.01 | 110.97 | 66427.67 |
| APOL2 | 24634.79 | 322.77 | 4255.49 | 108.89 | 5591.65 | 554.28 | 48058.26 |
| APOL3 | 3723.48 | 203.8 | 134.21 | 14.75 | 107.71 | 5.85 | 9806.32 |
| APOL4 | 4497.1 | 230.9 | 39.87 | 10.96 | 98.71 | 24.15 | 17712.7 |
| APOL6 | 29955.54 | 1036.07 | 159.36 | 9.02 | 646.85 | 41.64 | 34456.69 |
| APOM | 2917.58 | 118.1 | 2094.3 | 96.5 | 5945.29 | 1050.2 | 119652.97 |
| APOO | 3822.67 | 272.07 | 22267.35 | 1100.28 | 22672.12 | 2164.37 | 5539.33 |
| Values for mRNAs analyzed in this study are given as fold β-actin mRNA amounts multiplied by 10^-7^.Abbreviations: a: mRNA; b: hDF6 Mean; c: hDF6 SEM; d: WK6-iPSCs Mean; e: WK6-iPSCs SEM; f: WK6-HLCs Mean; g: WK6-HLCs SEM; h: Liver; SEM – Standard error of the mean | | | | | | | |
