# Supplementary material for: Cholesterol-Secreting and Statin-Responsive Hepatocytes from Human ES and iPS Cells to Model Hepatic Involvement in Cardiovascular Health
Source: PLoS One. 2013 Jul 11;8(7):e67296. doi: 10.1371/journal.pone.0067296 (PMC3708950; doi:10.1371/journal.pone.0067296)
Supplement: Table S2 — Regulation of gene expression for selected genes during hepatic differentiation of WA09 hES cells. (DOCX) [file pone.0067296.s004.docx]

| **Table S2. Regulation of gene expression for selected genes during hepatic differentiation of WA09 hES cells.** | | | | | |
| --- | --- | --- | --- | --- | --- |
| a | b | c | d | e | f |
| AFP | 2.69 | 2.69 | 276937.74 | 144711.57 | 97.71 |
| ALB | 3.89 | 3.89 | 1638.82 | 917.18 | 19056629.82 |
| CYP2E1 | 3.24 | 3.24 | 109.9 | 11.09 | 63554752.01 |
| GATA6 | 15.48 | 2.49 | 2338.67 | 1155.35 | 11405.93 |
| GHR | 432.5 | 80.64 | 4810.14 | 1658.55 | 203757.93 |
| GSTA1 | 111.81 | 19.75 | 6112.95 | 1741.85 | 13076713.49 |
| HMGCR | 139673.64 | 2698.58 | 112107.18 | 3428.44 | 26680.47 |
| HNF4A | ND | ND | 136.46 | 44.52 | 450602.77 |
| IGF1 | ND | ND | 654.06 | 176.66 | 10914.11 |
| IGF2 | 109.84 | 21.38 | 1186250.87 | 601011.5 | 283796.38 |
| IGFBP2 | 204369.19 | 10817.37 | 1523211.44 | 244744.87 | 677798.38 |
| LDLR | 12262.51 | 666.34 | 9035.49 | 838.04 | 79711.3 |
| MDR3 | 168.24 | 23.3 | 1720.04 | 376.19 | 143879.02 |
| POU5F1 | 1292187.21 | 95038.83 | 47711.13 | 11803.83 | 44777.82 |
| ZFP42 | 38229.82 | 3562.91 | 3499.82 | 948.12 | ND |
| RXRA | 3300.48 | 270.33 | 6165.2 | 707.62 | 432546.79 |
| SCARB1 | 9460.66 | 698.75 | 15325.13 | 3950.78 | 163337.52 |
| VIM | 116072.98 | 12986.62 | 1997610.61 | 52500.56 | 231796.08 |
| APOA1 | 147.22 | 41.36 | 238649 | 86977.16 | 17912913.4 |
| APOA2 | 2555.1 | 199.43 | 126082.28 | 46441.9 | 37243792.79 |
| APOA4 | ND | ND | 22729.71 | 12062.87 | 15842.33 |
| APOA5 | ND | ND | ND | ND | 129671.03 |
| APOB | 43.22 | 4.01 | 2592.94 | 1257.73 | 1083671.95 |
| APOC1 | 23794.2 | 1485.76 | 14748.36 | 4665.34 | 6341957 |
| APOC2 | 29.26 | 3.72 | 3370.79 | 1526.51 | 1876348.87 |
| APOC3 | 1.8 | 1.8 | 2769.99 | 1468.73 | 10224285.31 |
| APOC4 | ND | ND | ND | ND | 207895.16 |
| APOD | ND | ND | 246.65 | 116.83 | 554.67 |
| APOE | 186003.62 | 12136.61 | 157104.67 | 40114.65 | 6466244.66 |
| APOF | ND | ND | ND | ND | 128240.88 |
| APOH | 0.59 | 0.59 | 38.83 | 20.07 | 2326124.14 |
| APOL1 | 1213.11 | 77.25 | 1319.28 | 288.95 | 66427.67 |
| APOL2 | 6123.34 | 278.15 | 5697.81 | 1027.94 | 48058.26 |
| APOL3 | 167.56 | 89.44 | 143.06 | 23.79 | 9806.32 |
| APOL4 | 46.66 | 3.12 | 86.32 | 49.65 | 17712.7 |
| APOL6 | 111.72 | 21.05 | 625.08 | 128.34 | 34456.69 |
| APOM | 2491.06 | 109.47 | 9241.77 | 1623.9 | 119652.97 |
| APOO | 17978.76 | 972.84 | 15572.37 | 1019.38 | 5539.33 |
| Values for mRNAs analyzed in this study are given as fold β-actin mRNA amounts multiplied by 10^-7^.  Abbreviations: a: mRNA; b: WA09-ESCs Mean; c: WA09-ESCs SEM; d: WA09-HLCs Mean; e: WA09-HLCs SEM; f: Liver; SEM – Standard error of the mean | | | | | |
