# Supplementary material for: Cholesterol-Secreting and Statin-Responsive Hepatocytes from Human ES and iPS Cells to Model Hepatic Involvement in Cardiovascular Health
Source: PLoS One. 2013 Jul 11;8(7):e67296. doi: 10.1371/journal.pone.0067296 (PMC3708950; doi:10.1371/journal.pone.0067296)
Supplement: Table S3 — Regulation of gene expression for selected genes during hepatic differentiation of WK1 iPS cells derived from hDF1 fibroblasts. (DOCX) [file pone.0067296.s005.docx]

| **Table S3. Regulation of gene expression for selected genes during hepatic differentiation of WK1 iPS cells derived from hDF1 fibroblasts.** | | | | | | | |
| --- | --- | --- | --- | --- | --- | --- | --- |
| a | b | c | d | e | f | g | h |
| AFP | 21.74 | 21.74 | 2.62 | 2.62 | 34472.47 | 8358.59 | 97.71 |
| ALB | 2.02 | 2.02 | 3.51 | 0.34 | 270.84 | 63.79 | 19056629.82 |
| CYP2E1 | 324.97 | 108.41 | 3.01 | 1.63 | 61.59 | 5.11 | 63554752.0 |
| GATA6 | 145.65 | 58.94 | 205.03 | 36.87 | 1156.21 | 98.01 | 11405.93 |
| GHR | 1141.17 | 86.47 | 303.05 | 43.99 | 1225.99 | 211.08 | 203757.93 |
| GSTA1 | ND | ND | 104.7 | 21.36 | 3532.47 | 2245.97 | 13076713.5 |
| HMGCR | 10850.84 | 60.17 | 106255.83 | 10205.9 | 79843.26 | 3457.42 | 26680.47 |
| HNF4A | ND | ND | 1.32 | 0.67 | 83.54 | 3.92 | 450602.77 |
| IGF1 | 13.7 | 2.8 | 2.78 | 1.68 | 387.63 | 62.74 | 10914.11 |
| IGF2 | 40114.98 | 5348.04 | 137.94 | 31.71 | 243868.77 | 27382.84 | 283796.38 |
| IGFBP2 | 8526.74 | 909.13 | 160759.29 | 4936.51 | 577315.66 | 50921.05 | 677798.38 |
| LDLR | 7910.43 | 2192.67 | 12674.67 | 1480.25 | 8016.96 | 123.48 | 79711.3 |
| MDR3 | 77.48 | 11.53 | 244.05 | 16.6 | 1152.18 | 235.5 | 143879.02 |
| POU5F1 | 6247.34 | 715.68 | 1291597.71 | 85735.7 | 32267.05 | 8029.92 | 44777.82 |
| ZFP42 | ND | ND | 29422.44 | 2782.58 | 3169.01 | 164.63 | ND |
| RXRA | 5071.62 | 1596.88 | 3007.77 | 102.02 | 5113.22 | 255.3 | 432546.79 |
| SCARB1 | 4607.75 | 1157.85 | 11467.42 | 913.62 | 5677.23 | 475.86 | 163337.52 |
| VIM | 3546637.96 | 584635.68 | 109323.04 | 6998.24 | 1551061.58 | 126512.34 | 231796.08 |
| APOA1 | 16.42 | 8.63 | 2018.13 | 435.18 | 122555.18 | 16160.27 | 17912913.4 |
| APOA2 | 206.16 | 44.48 | 1214.48 | 318.76 | 46905.69 | 4549.63 | 37243792.79 |
| APOA4 | ND | ND | 11.58 | 5.85 | 3271.67 | 616.06 | 15842.33 |
| APOA5 | ND | ND | ND | ND | ND | ND | 129671.03 |
| APOB | 1.71 | 1.71 | 36.72 | 3.05 | 260.58 | 20.94 | 1083671.95 |
| APOC1 | 33 | 17.25 | 17546.97 | 2339.46 | 5202.67 | 643.88 | 6341957 |
| APOC2 | ND | ND | 18.41 | 3.63 | 392.6 | 58.21 | 1876348.87 |
| APOC3 | ND | ND | 2.48 | 2.48 | 982.73 | 312.81 | 10224285.31 |
| APOC4 | ND | ND | ND | ND | ND | ND | 207895.16 |
| APOD | 37586.09 | 13095.06 | 5.9 | 5.9 | 1408.64 | 208.45 | 554.67 |
| APOE | 200.97 | 81.12 | 154261.43 | 5059.78 | 53544.93 | 3728.84 | 6466244.66 |
| APOF | ND | ND | ND | ND | ND | ND | 128240.88 |
| APOH | ND | ND | 1.53 | 1.53 | 36.88 | 12.6 | 2326124.14 |
| APOL1 | 13115.95 | 553.06 | 909.76 | 112.86 | 1156.5 | 88.32 | 66427.67 |
| APOL2 | 23108.42 | 698.85 | 4190.27 | 210.65 | 5484.82 | 418.81 | 48058.26 |
| APOL3 | 3099.14 | 32.08 | 92.14 | 3.94 | 127.98 | 12.55 | 9806.32 |
| APOL4 | 3637.02 | 80.17 | 31 | 5.11 | 178.4 | 20.94 | 17712.7 |
| APOL6 | 30737.92 | 764.98 | 59.33 | 12.96 | 689.55 | 72.39 | 34456.69 |
| APOM | 2168.95 | 291.62 | 2126.47 | 125.06 | 4605.9 | 598.38 | 119652.97 |
| APOO | 2996.82 | 264.18 | 19543.25 | 1088.22 | 32371.5 | 2604.93 | 5539.33 |
| Values for mRNAs analyzed in this study are given as fold β-actin mRNA amounts multiplied by 10^-7^. Abbreviations: a: mRNA; b: hDF1 Mean; c: hDF1 SEM; d: WK1-iPSCs Mean; e: WK1-iPSCs SEM; f: WK1-HLCs Mean; g: WK1-HLCs SEM; h: Liver; SEM – Standard error of the mean | | | | | | | |
