# Supplementary material for: Cholesterol-Secreting and Statin-Responsive Hepatocytes from Human ES and iPS Cells to Model Hepatic Involvement in Cardiovascular Health
Source: PLoS One. 2013 Jul 11;8(7):e67296. doi: 10.1371/journal.pone.0067296 (PMC3708950; doi:10.1371/journal.pone.0067296)
Supplement: Table S4 — Regulation of gene expression for selected genes during hepatic differentiation of WK6 iPS cells derived from hDF6 fibroblasts. (DOCX) [file pone.0067296.s006.docx]

| **Table S4. Regulation of gene expression for selected genes during hepatic differentiation of WK6 iPS cells derived from hDF6 fibroblasts.** | | | | | | | |
| --- | --- | --- | --- | --- | --- | --- | --- |
| a | b | c | d | e | f | g | h |
| AFP | ND | ND | 1.17 | 0.59 | 85011.88 | 36140.18 | 97.71 |
| ALB | 2.9 | 2.9 | 2.53 | 0.63 | 378.75 | 148.07 | 19056629.82 |
| CYP2E1 | 466.35 | 8.31 | ND | ND | 87 | 21.14 | 63554752.01 |
| GATA6 | 17867.38 | 587.71 | 917.71 | 90.85 | 1649.32 | 646.18 | 11405.93 |
| GHR | 2374.43 | 313.02 | 371.78 | 23.9 | 3132.84 | 468 | 203757.93 |
| GSTA1 | ND | ND | 296.38 | 14.72 | 2852.21 | 1282.2 | 13076713.49 |
| HMGCR | 13477.82 | 561.54 | 79246.08 | 3119.68 | 60179.92 | 644.71 | 26680.47 |
| HNF4A | ND | ND | 4.8 | 0.33 | 134.28 | 58 | 450602.77 |
| IGF1 | 1836.89 | 424.55 | 0.63 | 0.63 | 702.06 | 113.64 | 10914.11 |
| IGF2 | 257.78 | 74.14 | 114.15 | 10.04 | 563589.38 | 47333.45 | 283796.38 |
| IGFBP2 | 163.84 | 37.39 | 132473.09 | 1468.45 | 429994.94 | 18682.21 | 677798.38 |
| LDLR | 11451.43 | 1259.32 | 10001.46 | 317.49 | 8016.22 | 923.3 | 79711.3 |
| MDR3 | 379.49 | 57.69 | 148.76 | 36.87 | 829.99 | 173.78 | 143879.02 |
| POU5F1 | 6841.93 | 396.93 | 1175284.2 | 79935.15 | 24395.33 | 2060.62 | 44777.82 |
| ZFP42 | ND | ND | 29741.49 | 2266.31 | 4052.92 | 289.1 | ND |
| RXRA | 7427.21 | 341.37 | 2574.29 | 124.02 | 4676.36 | 71.36 | 432546.79 |
| SCARB1 | 3397.17 | 136.48 | 8287.23 | 476.94 | 5606.01 | 460.81 | 163337.52 |
| VIM | 2269342.39 | 215472.43 | 94132.81 | 7569.89 | 1080069.59 | 114732.62 | 231796.08 |
| APOA1 | 2.16 | 2.16 | 383.93 | 54.61 | 118772.7 | 53018.68 | 17912913.4 |
| APOA2 | 217.62 | 46.11 | 2381.94 | 237.82 | 66089.52 | 27530.87 | 37243792.79 |
| APOA4 | ND | ND | ND | ND | 5535.89 | 2736.64 | 15842.33 |
| APOA5 | ND | ND | ND | ND | ND | ND | 129671.03 |
| APOB | ND | ND | 29.14 | 8.31 | 649.72 | 255.01 | 1083671.95 |
| APOC1 | 174 | 20.52 | 19840.2 | 1711.31 | 7152.69 | 1706.58 | 6341957 |
| APOC2 | ND | ND | 8.74 | 4.17 | 981.64 | 405.41 | 1876348.87 |
| APOC3 | ND | ND | ND | ND | 985.85 | 404.2 | 10224285.31 |
| APOC4 | ND | ND | ND | ND | ND | ND | 207895.16 |
| APOD | 14373.43 | 1040.89 | 6.09 | 3.05 | 972.6 | 406.46 | 554.67 |
| APOE | 1928.59 | 46.58 | 146907.45 | 7906.73 | 88533.68 | 5275.19 | 6466244.66 |
| APOF | ND | ND | ND | ND | ND | ND | 128240.88 |
| APOH | ND | ND | 2.17 | 2.17 | 28.48 | 14.49 | 2326124.14 |
| APOL1 | 9607.99 | 467.25 | 941.44 | 150.74 | 1334.01 | 110.97 | 66427.67 |
| APOL2 | 24634.79 | 322.77 | 4255.49 | 108.89 | 5591.65 | 554.28 | 48058.26 |
| APOL3 | 3723.48 | 203.8 | 134.21 | 14.75 | 107.71 | 5.85 | 9806.32 |
| APOL4 | 4497.1 | 230.9 | 39.87 | 10.96 | 98.71 | 24.15 | 17712.7 |
| APOL6 | 29955.54 | 1036.07 | 159.36 | 9.02 | 646.85 | 41.64 | 34456.69 |
| APOM | 2917.58 | 118.1 | 2094.3 | 96.5 | 5945.29 | 1050.2 | 119652.97 |
| APOO | 3822.67 | 272.07 | 22267.35 | 1100.28 | 22672.12 | 2164.37 | 5539.33 |
| Values for mRNAs analyzed in this study are given as fold β-actin mRNA amounts multiplied by 10^-7^. Abbreviations: a: mRNA; b: hDF6 Mean; c: hDF6 SEM; d: WK6-iPSCs Mean; e: WK6-iPSCs SEM; f: WK6-HLCs Mean; g: WK6-HLCs SEM; h: Liver; SEM – Standard error of the mean | | | | | | | |
